# Integrated Authentication Server Design for Efficient Kerberos–Blockchain VANET Authentication †

**DOI:** 10.3390/s25216651

**Published:** 2025-10-30

**Authors:** Maya Rahayu, Md. Biplob Hossain, Samsul Huda, Yasuyuki Nogami

**Affiliations:** 1 Graduate School of Environmental, Life, Natural Science and Technology, Okayama University, Okayama 700-8530, Japan; p8rg1uci@s.okayama-u.ac.jp (M.B.H.); yasuyuki.nogami@okayama-u.ac.jp (Y.N.); 2 Electrical Engineering Departement, Politeknik Negeri Bandung, Bandung 40559, Indonesia; 3 Electrical and Electronic Engineering Department, Khwaja Yunus Ali University, Sirajganj 6751, Bangladesh; 4 Interdisciplinary Education and Research Field, Okayama University, Okayama 700-8530, Japan; shuda@okayama-u.ac.jp

**Keywords:** VANET security, blockchain, integrated authentication server, Kerberos authentication, Vehicular Ad Hoc Network

## Abstract

Vehicular Ad Hoc Network (VANET) is a fundamental component of the intelligent transportation systems (ITS), providing critical road information to users. However, the volatility of VANETs creates significant vulnerabilities from malicious actors. Thus, verifying joining entities is crucial to maintaining the VANET’s communication security. Authentication delays must stay below 100 ms to meet VANET requirements, posing a major challenge for security. Our previous research introduced a Kerberos–Blockchain (KBC) authentication system that contains two main components separately: Authentication Server (AS) and Ticket Granting Server (TGS). However, this KBC architecture required an additional server to accommodate increasing vehicle volumes in urban environments, leading to higher infrastructure costs. This paper presents an integrated authentication server that merges AS and TGS into a Combined Server (CBS) while retaining blockchain security. We evaluate it using OMNeT++ with SUMO for traffic simulation and Ganache for blockchain implementation. Results show that CBS removes the need for an extra server while keeping authentication delays under 100 ms. It also improves throughput by 104% and reduces signaling overhead by 45% compared to KBC. By optimizing authentication without compromising security, the integrated server greatly enhances the cost-effectiveness and efficiency of VANET systems.

## 1. Introduction

The intelligent transportation systems (ITS) contributes to the development of a sophisticated transport network in the era of Digital Transformation (DX) [1]. A crucial aspect of the ITS is its ability to facilitate communication between vehicles and roadway infrastructure, commonly referred to as Vehicular Ad Hoc Network (VANET). The primary objective of such communication is to articulate critical messages that encompass various elements including speed, geographical position, trajectory, and urgent alerts that signify hazardous conditions [2].

The potential inherent in these capabilities lies in the augmentation of efficiency and the regulation of vehicular movement by disseminating real-time information to vehicles, thereby refining their navigational paths and alleviating traffic obstruction [3]. The supervision of traffic flow through the distribution of real-time information to vehicles, optimizing their routes, and mitigating traffic congestion [3].

**Figure 1:**
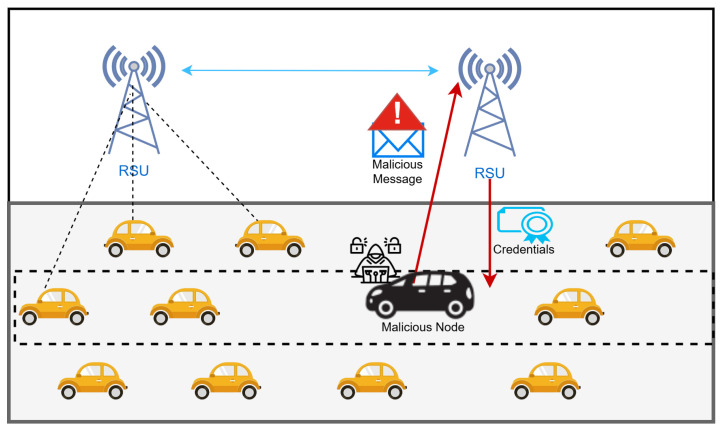
VANET’s vulnerability, adapted from our previous work [8].

However, despite these promising capabilities, the volatile architecture of VANETs—characterized by high node mobility and frequently changing topology—introduces significant vulnerabilities within the network. As shown in Figure 1, the illustration highlights the potential security risks in VANETs due to their highly dynamic and volatile nature. An adversary can exploit these vulnerabilities by either intercepting sensitive data transmitted between vehicles and Roadside Units (RSUs), or by injecting malicious messages into the network, posing serious threats to network integrity and vehicular safety.

The establishment of a robust authentication protocol within VANETs is essential to impede the transmission of malevolent messages by adversaries and to protect vital network information [4]. Failing to execute this validation may allow for unapproved vehicles to propagate erroneous information or engage in nefarious activities without detection. The authentication time in VANETs is necessary for its security. A significant delay in the authentication process may result in the utilization of outdated or unreliable information for driving decisions, which could potentially precipitate accidents. Consequently, it is imperative to establish an effective mechanism for the authentication of incoming messages under a limited temporal constraint of 100 ms, preceding the distribution of a new safety alert [5].

In previous research, we made an authentication system that utilized the Kerberos–Blockchain (KBC) authentication method. KBC includes two main modules, AS and TGS, separately. This method supported the security both maintaining the authentication time. In [6], the authors focused on implementing the Kerberos authentication protocol within a VANET environment. In [7], the authors integrated Kerberos authentication with blockchain technology, where blockchain stores the authentication messages generated from Kerberos authentication to support the handover phase. Furthermore, in [8], this system was explained deeper, evaluating several VANET scenarios, including both suburban and urban areas.

The findings of this research indicate that the system meets the authentication delay requirements when the number of vehicles is 100. However, when the vehicle count reaches 200, an additional Kerberos server is required to maintain the authentication delay requirements; otherwise, it will exceed the authentication delay requirement for VANETs.

However, this architectural expansion to add more servers also introduces a range of financial and operational implications. From the financial perspective, the need for multiple servers increases the overall infrastructure cost, both for hardware and software licenses. In terms of system design, an increased number of KBC servers can cause the complexity because it requires careful coordination and load-balancing to ensure the synchronization of authentication data across all nodes.

In this study, we propose an optimization of the Kerberos–Blockchain-based authentication scheme by combining the Authentication Server (AS) and Ticket Granting Server (TGS) into a single Combined Server (CBS). This streamlines the authentication process to reduce overall authentication delay by reducing the number of interactions required during the authentication phase. We utilized blockchain to record the authentication messages generated by Kerberos authentication to reduce the handover delay while also maintaining security. The system utilized the Advanced Encryption Standard (AES) with a 128-bit key and employs the Ethereum blockchain.

To prove our proposed system’s effectiveness, we assessed parameters including authentication delay, throughput, and signaling overhead. This evaluated the simulation environments, divided into on-chain and off-chain. In the on-chain environment, Ganache is used for blockchain simulation, alongside Truffle for smart contract management. Meanwhile, the off-chain environment utilizes OMNeT++ for network simulation and SUMO for modeling vehicular mobility in VANET scenarios.

To clarify the novelty of our Combined Server (CBS) design, we emphasize that this architecture is not a mere colocation of the Authentication Server (AS) and Ticket Granting Server (TGS), but rather a protocol-aware optimization that fundamentally restructures the Kerberos authentication flow. This integration reduces authentication rounds, lowers latency, and consolidates processing load, resulting in faster, more scalable authentication while preserving the original Kerberos trust model.

Unlike existing Kerberos–Blockchain schemes, our Combined Server architecture introduces protocol-level innovations that streamline authentication by collapsing multi-step Kerberos exchanges and enabling blockchain-supported session reuse during RSU handovers.

The contributions of this manuscript are delineated as follows:

- A unified Kerberos Server architecture that eliminates the need for separate AS and TGS components while maintaining authentication delays below the critical 100 ms threshold for Efficient Kerberos–Blockchain VANET Authentication.
- Comprehensive evaluation using Omnet++ network simulator integrated with SUMO traffic simulator to model both suburban and urban scenarios, with blockchain components implemented on the Ganache platform.
- Quantitative performance analysis shows a 104% increase in system throughput and a 45% reduction in signaling overhead compared to the conventional separated-server architecture [8].

## 2. Literature Study

The dynamic and distributed nature of VANETs presents significant authentication challenges, necessitating the development of efficient and secure identity verification protocols. Various approaches have been proposed, such as the Two-Factor Lightweight Privacy-preserving (2FLIP) scheme, which integrates biometric-password-based authentication with decentralized certificate authorities to reduce computational costs and protect user privacy [9]. Similarly, the Secure and Efficient Message Authentication (SEMA) protocol utilizes a hybrid pseudonym and group-oriented methodology to facilitate reciprocal authentication between vehicles and Road Side Units (RSUs), aiming to preserve privacy while preventing false accusations and malicious behaviors [10]. Another relevant study introduces a vehicle-to-vehicle authentication mechanism incorporating password update strategies and honey list defenses to counter offline guessing attacks [11]. Despite their contributions, these solutions still rely on centralized architectures, leaving them vulnerable to single points of failure and potential data manipulation threats.

To overcome the limitations of centralized authentication in VANETs—such as scalability issues and single points of failure—recent research has increasingly adopted blockchain-based architectures to enhance trust and decentralization. By leveraging immutable ledgers and distributed consensus mechanisms, these frameworks improve the integrity and reliability of authentication processes in dynamic vehicular environments. For instance, the work in [12] integrates the InterPlanetary File System (IPFS), Ciphertext-Policy Attribute-Based Encryption (CP-ABE), and Ethereum blockchain to ensure secure and decentralized data management in VANETs. Similarly, a blockchain-based authentication infrastructure proposed in [13] utilizes AES, TKIP, and CCMP cryptographic protocols in conjunction with Hyperledger Fabric to securely manage user credentials and facilitate trustless authentication across network nodes.

Some researches also implemented blockchain technology that combined with Kerberos authentication to support the security both maintaining the authentication time. In [6], the investigation concentrates on the application of the Kerberos authentication protocol within the context of a VANET environment. In [7], the authors work combined Kerberos authentication with blockchain technology, wherein the blockchain serves as a repository for the authentication messages generated by the Kerberos authentication process to facilitate the handover phase. Furthermore, in [8], the system is elucidated in greater detail, assessing various VANET scenarios that encompass both suburban and urban locales.

Recent studies have explored integrating security and QoS into VANET authentication frameworks. In [14], the authors proposed a secure, RFID-based authentication mechanism using a toll-oriented certificate management system. Their design emphasized secure V2I and I2V communications while introducing a mobility-aware node attachment strategy to fulfill QoS requirements. Similarly, Ref. [15] presented a generation certification-based vehicle authentication protocol using RFID tags and readers, complemented by a QoS-aware mobility management scheme to ensure both reliable communication and data privacy.

While these works offer valuable contributions, they primarily rely on physical-layer mechanisms (e.g., RFID) and lack scalable, trust-enhanced infrastructure for broader VANET ecosystems. Our proposed CBS framework builds upon these foundations by transitioning toward a blockchain-integrated architecture. Unlike RFID-based schemes, CBS leverages decentralized consensus, encrypted authentication messages, and smart contract-enforced access control, thereby improving scalability, auditability, and security guarantees. Moreover, CBS is designed to support multi-entity interactions and efficient authentication workflows without sacrificing privacy or system availability—addressing some of the deployment and trust limitations observed in earlier RFID-based designs.

In [16], the authors proposed a blockchain-based framework for secure and privacy-preserving sharing of car location information in the online ride-hailing industry. The system integrates homomorphic encryption and probabilistic verification to enable selective disclosure and batch verification of location data, ensuring both privacy and data authenticity. A novel hash-exclusive-or tree structure was employed to minimize computational overhead, outperforming traditional Merkle tree schemes, especially in high-volume data scenarios. The experimental evaluation demonstrated microsecond-level time and kilobyte-level storage efficiency under a 128-bit security level, making the approach suitable for high-throughput vehicular data sharing.

This work aligns conceptually with our proposed CBS framework, as both aim to preserve user privacy while leveraging blockchain to secure vehicular data. While [16] focuses on the selective disclosure of location data in ride-hailing services, our system targets authentication and access control in VANETs. The CBS architecture utilizes AES encryption and smart contracts to secure authentication messages, incorporating a Kerberos-like mechanism and off-chain message handling to reduce latency and blockchain load. In contrast, Ref. [16] emphasizes data sharing efficiency via homomorphic encryption and hash-XOR trees. Despite the differences in focus and cryptographic techniques, both systems demonstrate effective privacy preservation and blockchain-based scalability for secure vehicular applications.

Table 1 summarizes the key features of recent VANET authentication proposals. Several works use traditional cryptography without blockchain. In [17], authors present 2FLIP, a lightweight, privacy-preserving VANET authentication scheme using decentralized CA and biometric 2FA, achieving lower computation and 55–77% less communication cost, with strong privacy, DoS resistance, and support for real-time emergency reporting. The main limitation of 2FLIP lies in its dependence on a single CA-managed system key, which, if compromised, could threaten overall VANET security despite partial decentralization to OBUs and telematics devices.

A hybrid message authentication protocol that effectively addresses challenges related to pseudonym-based and group-based authentication methods in VANETs was proposed in [18]. An efficient and secure group-based message authentication algorithm is designed specifically for V2V communication, ensuring that it is framing-free to prevent valid vehicles from being framed by compromised entities.

SEMA relies on a fully trusted central authority, incurs pairing computation overhead, and lacks efficient revocation and real-world validation for large-scale VANET deployment.

The authors proposed a blockchain-based decentralized pseudonym management scheme for VANETs in [19] that shifts authentication tasks from vehicles to RSUs, reducing computational load and authentication delay. It achieves about 47–50% lower delay and smaller packet sizes compared to traditional PKI schemes, while maintaining conditional anonymity and accountability. However, the scheme increases message exchanges with RSUs, causing higher channel congestion under heavy traffic and lacks real-world testbed validation for scalability.

Meanwhile, Ref. [20] employed a consortium blockchain-based trust management framework that enables vehicles to request location-based services without revealing sensitive identity or location information by using anonymous cloaking regions and trust evaluation. They also integrated homomorphic encryption and blockchain storage for secure trust value computation and updates, thereby enhancing both the privacy preservation and trustworthiness of vehicular communications. The scheme still incurs computational and communication, which may limit real-time scalability in dense VANET scenarios.

A more recent roaming-auth protocol, BARA [21], proposes a blockchain-based anonymous authentication scheme for VANET traffic, reporting that integrates certificateless message authentication, adaptive threshold multi-signature, and aggregation verification. The authors also introduced a blockchain-based TCoin incentive mechanism to ensure authenticity, anonymity, and active participation in traffic reporting. However, the scheme depends on RSUs for consensus and incurs high computational overhead from bilinear pairing operations, limiting scalability in large-scale or real-time VANET scenarios.

In addition, EBAS [22] presents an efficient blockchain-based authentication scheme for secure VANET communication, combining UTXO-based transaction validation and ECC-based pseudonyms to ensure anonymous, scalable, and fast authentication. It introduces a transaction update mechanism to maintain stable storage and low retrieval overhead, achieving an average authentication cost of 0.942 ms, outperforming existing schemes. However, EBAS depends on regional trusted authorities (RTAs) for authentication and blockchain maintenance, introducing centralized trust and potential bottlenecks under large-scale VANET conditions.

**Table 1:** Comparative study of the proposed literature with existing authentication protocols for VANETs.

Reference/ Year	Authentication Protocol	Blockchain Use	Server Architecture	Simulation Tool	Perf. Metrics Evaluated	Key Findings
[17]/2015	2FLIP (Two-Factor Lightweight Privacy-Preserving)	No	Centralized CA + RSU	NS-2	Auth. delay, packet loss	Near-zero auth delay and 0% packet loss via decentralized CA and biometric 2FA
[18]/2021	SEMA (pseudonym + group authentication)	No	Centralized RSU-based CA	SUMO + NS-3	Mutual authentication	Achieves vehicle–RSU mutual auth while preserving privacy
[19]/2021	Blockchain-based pseudonym management	Yes (permissioned)	Pseudonym CA + RSUs	SUMO + OMNeT++	Auth. delay, OBU computation	47% reduction in auth delay and OBU computation; extra RSU signaling
[20]/2023	Privacy-preserving trust management	Yes (public blockchain)	RSU	Custom simulation	Trust accuracy, malice detection	Blockchain stores trust scores; RSUs verify reports anonymously
[21]/2025	BARA (Blockchain-based Anonymous Roaming Auth.)	Yes	Decentralized, no central server	Scyther + BAN logic	Comm./comp. cost, revocation	Ensures anonymity and forward secrecy; reduces on-chain storage
[22]/2022	EBAS (ECC + Hyperledger Fabric)	Yes (Fabric)	Consortium blockchain	Hyperledger Fabric, NS-2	Auth. overhead, storage cost	Avg. auth. overhead 0.94 ms; outperforms existing schemes
[6]/2023	Kerberos + Blockchain integration	Yes (Ethereum)	AS + TGS, RSUs	OMNeT++, SUMO	Auth. delay, signaling overhead	Auth delay 63–95 ms up to 100 vehicles with minimal overhead
[8]/2024	Kerberos+Blockchain (KBC) in diverse scenarios	Yes (Ethereum)	AS + TGS, RSUs, Ethereum blockchain	OMNeT++, SUMO, Ganache, Truffle	Auth. delay, handover delay, end-to-end delay	Within 100 ms delay for 100 vehicles; 200 vehicles need extra KBC; gas grows linearly
**Proposed Work/2025**	Combined Server (CBS) of Kerberos+Blockchain	Yes (Ethereum)	Combined Server, RSUs, Ethereum blockchain	OMNeT++, SUMO, Ganache, Truffle	Auth. delay, signalling overhead, throughput	Meets 100 ms auth delay until 300 vehicles; higher throughput and lower overhead than KBC

In contrast, only a few efforts combine Kerberos with blockchain. In our previous work [6], we built a Kerberos-based scheme with a blockchain ledger accessible to a KBC and RSUs. The simulation, comprising 100 vehicles and 4 RSUs, demonstrated low delays ranging from 63 to 95 ms and minimal signaling overhead. Similarly, in another work [8], we evaluated the KBC authentication approach under suburban and urban scenarios, finding that it was viable (meeting delay and throughput requirements) even as server count or vehicle density increased. However, these studies still employed separate AS and TGS entities inside the KBC server.

Overall, the prior literature reveals that while blockchain can enhance privacy and trust, and Kerberos can provide strong mutual authentication; the combination of the two has not yet been explored in a unified-server setting. Most existing Kerberos–Blockchain schemes maintain multiple servers and do not fully address signaling cost in dense networks. The proposed approach differs by merging the AS and TGS functionalities into a single server, thereby reducing protocol rounds and directly filling the gap of minimizing theauthentication overhead identified in earlier studies.

## 3. Preliminaries

This section presents a theoretical analysis of Vehicular Ad Hoc Networks (VANETs), a detailed explanation of the Kerberos authentication mechanism, and the blockchain framework utilized in the current investigation. Furthermore, this subsection discusses, in detail, the secure authentication protocols used in VANETs.

### 3.1. Vehicular Ad Hoc Network

VANETs are decentralized wireless networks in which vehicles communicate with each other and with roadside infrastructure in an ad hoc manner, without relying on fixed-base stations. As a key component of modern ITSs, VANETs aim to enhance road safety, optimize traffic flow, and provide real-time information dissemination [23]. Standard VANET architecture includes three core entities: On-Board Units (OBUs), Roadside Units (RSUs), and a server. OBUs, embedded in vehicles, are responsible for transmitting and receiving safety messages, while RSUs are fixed nodes deployed along roadways to facilitate vehicle-to-infrastructure (V2I) communication. These components interact using Dedicated Short Range Communication (DSRC) technologies, especially grounded in the IEEE 802.11p standard, which is alternatively referred to as Wireless Access in Vehicular Environments (WAVE) [24].

VANETs are characterized by high mobility, dynamic topology, real-time communication demands, and relatively strong computation capabilities due to the availability of in-vehicle resources. Vehicles frequently join and leave the network due to mobility, leading to rapidly changing network topologies. While energy consumption is less of a concern in VANETs compared to Mobile Ad Hoc Networks (MANETs), latency and reliability remain critical, particularly for safety applications. Traditionally, IEEE 802.11p has served as the standard protocol in VANETs, operating in the 5.9 GHz band to support low-latency vehicle-to-vehicle (V2V) and V2I communication.

### 3.2. Abbreviations and Acronyms

This subsection elucidates a range of annotations and abbreviations pertinent to all terminologies articulated within this manuscript, which include entities, keys, message designations, and their respective contents. The extensive compilation of abbreviations and annotations is delineated in Table 2.

### 3.3. Kerberos Authentication

The Kerberos employs symmetric key cryptography to establish a highly secure authentication framework for both vehicular units and Roadside Units (RSUs). The incorporation of a session key for vital operations, which include interactions with the Ticket-Granting Server (TGS) and specific services, distinguishes Kerberos authentication as a notable protocol within the field of cybersecurity. The decryption of messages that transmit session keys and other essential information is executed through the utilization of the confidential keys associated with the pertinent entities. The original authentication mechanism utilized in Kerberos V5 [25] can be delineated as follows:

(1) $$\begin{array}{cc} RA= & (ID_{v}||Realm_{v}||ID_{TGS}||Times||Nonce_{1}\left||PreAuth\right) \end{array}$$

(2) $$\begin{array}{c} RP=ID_{v}\;∥\;TGS\;∥\;E(K_{vs},[K_{TGS}\;∥\;\\ Times\;∥\;Nonce_{1}\;∥\;Realm_{TGS}\;∥\;ID_{TGS}]) \end{array}$$

### 3.4. Blockchain and Ethereum-Based Authentication

Blockchain is a distributed ledger technology that organizes transactions into blocks, which are cryptographically linked using hash pointers to form an immutable chain [26]. Each block contains a hash of the previous block’s contents, ensuring that any alteration in one block invalidates all subsequent blocks. This cryptographic integrity, combined with consensus algorithms such as Proof-of-Work or Proof-of-Stake, enables a decentralized trust model where no single entity controls the ledger. Once a transaction is validated and appended to the blockchain, it becomes tamper-resistant and verifiable by all participants in the network, making blockchain an ideal foundation for secure data exchange and verification.

Ethereum extends the capabilities of traditional blockchains by supporting decentralized applications (dApps) through its Turing-complete Ethereum Virtual Machine (EVM) [27]. Unlike Bitcoin’s limited scripting functionality, Ethereum enables smart contracts—self-executing programs deployed on-chain that autonomously enforce logic and agreements. These smart contracts are validated and replicated across all nodes in the network, ensuring transparency and deterministic execution. Ethereum initially employed a Proof-of-Work consensus mechanism and has since transitioned to Proof-of-Stake to enhance scalability and energy efficiency, making it increasingly suitable for real-world, high-volume applications.

Given these characteristics, Ethereum is especially advantageous for authentication systems in dynamic environments such as VANET and Internet of Things (IoT) applications. Recent studies have leveraged Ethereum smart contracts to provide decentralized, tamper-proof authentication services. For example, Ref. [28] proposed a blockchain-integrated IoT authentication framework that secures device credentials via Ethereum-based validation. Similarly, Ref. [29] developed a VANET authentication model called TR-Block, which utilizes Ethereum smart contracts to verify and distribute trustworthy vehicular data. These applications demonstrate Ethereum’s ability to enhance system security, reduce reliance on centralized authorities, and maintain low authentication latency in real-time communication scenarios.

## 4. Overview of Kerberos–Blockchain VANET Authentication with Separated AS and TGS (KBC)

Earlier Kerberos–Blockchain VANET Authentication (KBC) schemes [6,8] follow the classical Kerberos model, which includes separate AS and TGS components. The authentication messages produced by the KBC server are stored in the blockchain system. In this architecture, a Vehicle (V), Roadside Units (RSUs), and the KBC server interact through a sequence of steps.

First, V registers and obtains a long-term secret from the AS. During authentication, V sends an Authentication Request (RA) to the AS, including its ID, the desired RSU service, and a requested ticket lifetime. The AS replies with a Ticket-Granting Ticket (TGT) and associated keys. V then contacts the TGS by presenting the TGT and an authentication message. The TGS validates the request, generates a Service Ticket (ST) for the target RSU, and returns it to V.

**Figure 2:**
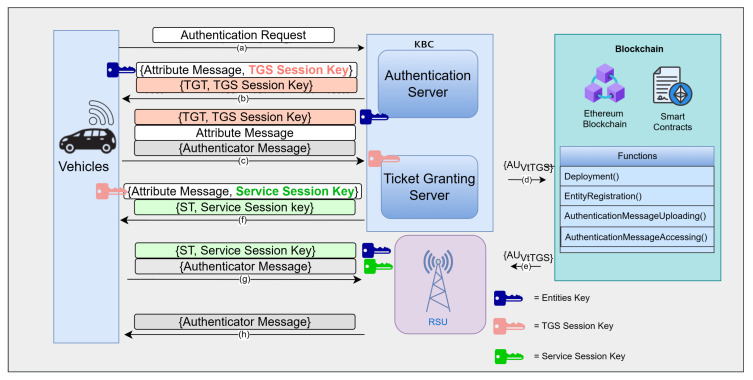
Kerberos-Blockchain VANET authentication phase with separated AS and TGS. (**a**) Vehicle sends authentication request to KBC; (**b**) AS verifies the vehicle and generates TGS session key and Ticket Granting Ticket (TGT) to the vehicle; (**c**) Vehicle sends request to TGS with the received TGS key and TGT; (**d**) TGS store the authentication message to the blockchain ledger; (**e**) Blockchain ledger send the authentication message to RSU; (**f**) TGS issues Service Ticket (ST) to the vehicle; (**g**) Vehicle presents ST to the RSU for service access; (**h**) RSU validates the ticket and grants service. authorization.

Crucially, the TGS stage also integrates blockchain: before issuing the ST, the TGS checks the incoming authentication message against the distributed ledger to ensure that it has not been seen before, and then appends it to the blockchain ledger. This provides immutable evidence of each authentication event, but does not alter the core Kerberos flow. After TGS response, V finally uses the service ticket to authenticate to the RSU. It sends (ST, authentication message) to the RSU, which decrypts the ticket, verifies mutual keys, and allows for V access to network services. The illustration of Kerberos–Blockchain VANET authentication phase with separated AS and TGS is shown in Figure 2.

Key findings from these studies include acceptable performance despite the extra blockchain step. The authentication delay remains under safety thresholds (e.g., rising from 63 ms to 95 ms as vehicles increase from 5 to 100). The signaling overhead analysis shows that, by merging some messages, Kerberos–Blockchain scheme had lower overhead than comparable designs. However, this separated KBC architecture has limitations. Under heavy loads or in urban deployments, the KBC servers exchange incurs latency and additional message exchanges, which can be costly. The dual-server setup also means doubling the required infrastructure and computational effort at both servers. Even though the KBC can be scaled, each additional server adds deployment cost.

These considerations motivate our integrated server design. By collapsing AS and TGS inside KBC into a single logical server, the protocol eliminates at least one message round and simplifies key management. In essence, our approach preserves the security benefits of Kerberos and blockchain while addressing the performance and scalability limitations identified in earlier works.

## 5. Proposed Integrated Authentication Server Design (CBS)

This section discuss the authentication method and authentication stages proposed in this research and the entities that influenced these stages. These stages include the initial authentication stage, the stage where authentication messages are stored in blockchain, and the handover stage.

### 5.1. Overview of the System

We utilized the blockchain to record the authentication messages generated by Kerberos authentication to reduce the handover delay while also maintaining the security. The proposed system integrates Kerberos authentication within a blockchain framework by recording authentication messages generated by the Kerberos server onto the blockchain. By leveraging the decentralized nature of blockchain technology, the system aims to reduce authentication delays, particularly during the handover process in vehicular networks.

The system is structured into three main stages: the initial authentication stage, where authentication messages are generated; the blockchain uploading stage, where these messages are recorded onto the blockchain; and the re-authentication stage, conducted during handovers. After the initial authentication is completed, the vehicle no longer requires a direct connection to the Kerberos server, as subsequent re-authentication processes only involve verifying authentication messages stored on the blockchain. This study focuses on optimizing the Kerberos authentication process by combining the Authentication Server (AS) and the Ticket Granting Server (TGS) to achieve lower authentication times compared to existing approaches.

**Figure 3:**
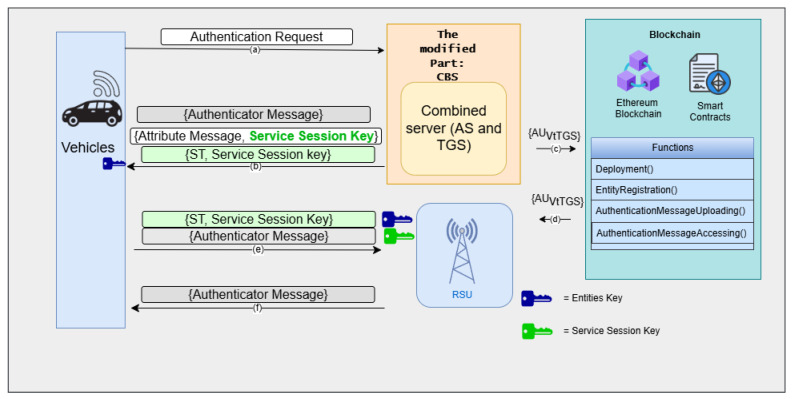
Proposed system architecture integrating AS and TGS into the combined blockchain server (CBS). (**a**) Vehicle sends authentication request to KBC; (**b**) AS verifies the vehicle and generates Service Ticket and Service Session Key to the vehicle; (**c**) TGS store the authentication message to the blockchain ledger; (**d**) Blockchain ledger send the authentication message to RSU; (**e**) Vehicle presents ST to the RSU for service access; (**f**) RSU validates the ticket and grants service.

The system utilized the Advanced Encryption Standard (AES) with a 128-bit key and is applied to secure the Kerberos authentication process. For the blockchain framework, the system employs the Ethereum blockchain. This proposed system figure is depicted in Figure 3.

In this study, we reuse the suburban and urban mobility scenarios introduced in our previous work [8], generated using SUMO–OMNeT++ with the Duarouter mobility model. However, we extend the evaluation in two key directions. First, we analyze the proposed CBS architecture under the same scenarios, but across multiple delay metrics: authentication delay, ticket validation delay, and blockchain transaction delay. Second, to assess the scalability of CBS under higher vehicle densities, we introduce additional simulation configurations with 300 and 400 vehicle nodes in the suburban scenario. These new simulation runs provide insights into CBS performance in more congested environments and allow for a more thorough comparison against the baseline architecture.

#### 5.1.1. Entities and Functions

Our system comprises three fundamental entities: the Combined Server (CBS) that has function as the Trusted Authentication Server, the Road Side Unit (RSU), and the vehicle. The CBS functions as a server responsible for registering all entities, authenticating vehicles, and disseminating authentication messages to the blockchain. RSUs serve as intermediaries between the vehicles and the CBS. They facilitate the integration of vehicles into the network, assist in the transfer of data among network entities, and disseminate information pertinent to traffic conditions. The vehicles assume the role of network nodes and data sources. Each vehicle is equipped with an On-board unit (OBU), enabling it to communicate road-related data to nearby RSUs and neighboring vehicles within its communication range.

#### 5.1.2. Overview of the Phases

The proposed system architecture is structured into three primary phases: system initialization and entity registration, initial authentication, and the handover procedure.

The registration stage serves the purpose of documenting all entities, which include the RSUs, CBS, and vehicles.

The initial authentication process is initiated when a vehicle enters the network and submits an authentication request to the CBS. Upon successful authentication, it issues a service ticket to the vehicle, granting it access to connect to the network via RSU1.

The CBS generates authentication messages, which are subsequently uploaded to the blockchain. These messages are utilized by vehicles during the handover process to facilitate seamless re-authentication.

When a vehicle moves beyond the coverage area of RSU1, it initiates a handover procedure by sending its previously issued authentication message to RSU2. RSU2 then interacts with the blockchain to retrieve and verify the corresponding credential stored during the initial authentication phase. If the provided credential matches the one recorded on the blockchain, the vehicle is successfully re-authenticated. This blockchain-assisted handover mechanism eliminates the need to repeat the initial authentication process, thereby significantly minimizing the overall authentication delay.

### 5.2. The Combination of the Server

In this research, we focuss on the combination of the Authentication Server (AS) and (TGS) from the conventional Kerberos–Blockchain system (KBS) to becomes the combined server (CBS) to reach higher performance without any additional infrastructure.

In the original modular architecture, the AS was responsible for handling vehicle registration, secret key issuance, and generating Authentication Messages, whereas the TGS handled ticket validation and Secure Ticket (ST) generation for service access. In contrast, the proposed CBS architecture consolidates these responsibilities into a single logical entity, allowing for more efficient and centralized handling of authentication processes.

A critical advantage of this unified design is the removal of inter-module communication. In the previous KBC model, packets such as authentication messages and session keys, needed to be transmitted between separate servers, introducing delays and processing overhead, especially under high vehicle density. By contrast, the CBS model eliminates these transmissions by directly processing all authentication steps within one server. For example, the socketDataArrived function in KBC now handles both ticket validation and secure ticket generation, which minimizes the need for inter-module signaling and improves overall efficiency.

Furthermore, the integration into a single server significantly improves end-to-end delay performance. In the KBC model, communication latency between the modules often led to processing bottlenecks, especially during handovers or in scenarios with rapid vehicle mobility. The CBS design, through centralized processing in functions such as handleRegistrationOfVehiclePacket and handleVehicleToTGSPacket, reduces these delays by enabling direct and immediate handling of all authentication-related packets.

The unified architecture also leads to a more efficient cryptographic key management scheme. In the previous model, AS and TGS each maintained their own sets of secret and session keys, increasing complexity and coordination requirements. In the CBS system, key generation and management—such as the generateSecretKey routine—are performed within a single scope. This centralization allows for simplified encryption workflows and secure ticket issuance, reducing the risk of synchronization issues between multiple servers.

Lastly, the CBS model offers considerable simplification of the overall codebase. The KBC architecture required distinct initialization, runtime operations, and teardown procedures, which increased software maintenance effort and redundancy. The CBS implementation reduces this complexity by utilizing unified methods such as initialize and a single event-handling function, socketDataArrived, to perform the complete authentication lifecycle. This makes the codebase more maintainable and extensible for future enhancements or deployment scenarios.

In contrast to prior Kerberos–Blockchain frameworks where the Authentication Server (AS) and Ticket Granting Server (TGS) were implemented as separate modules, our proposed architecture integrates these roles into a single Combined Server (CBS). This integration is a purposeful optimization to streamline the authentication process [6,8].

**Figure 4:**
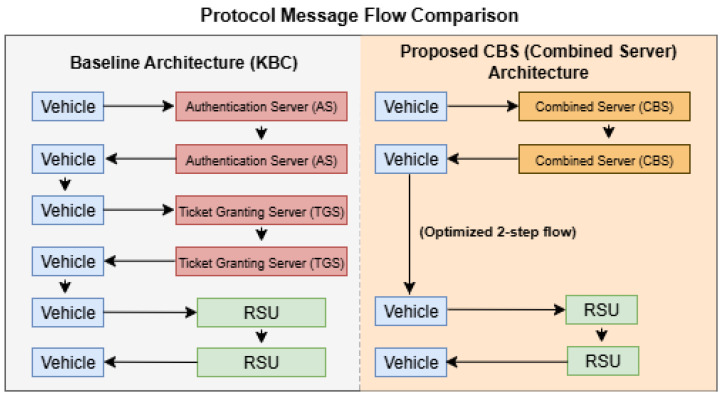
Protocol message flow comparison between baseline (KBC) and the proposed Combined Server (CBS) architecture. *Repeated entities are visualized to indicate each protocol step, although they represent a single logical instance*.

This integration is shown in the Figure 4. In the baseline Kerberos architecture, authentication messages must pass through two servers (AS and TGS) before reaching the RSU, resulting in four protocol steps. In contrast, the CBS architecture eliminates one server layer, reducing the flow to two major hops, CBS and RSU, thus minimizing communication latency.

First, merging AS and TGS eliminates the inter-server communication steps inherent in traditional Kerberos. In the separated design, each vehicle’s authentication requires two sequential exchanges (AS followed by TGS), incurring an extra network round-trip and processing delay. The unified CBS handles the entire ticket issuance in one place, reducing the number of message exchanges and thus lowering the overall authentication latency.

Second, the unified server architecture improves resource utilization and scalability through load consolidation. Instead of two servers that might individually run at partial load or become unevenly bottlenecked, the CBS serves a single aggregated request queue, ensuring full utilization of server capacity. This means that the system can handle higher vehicle volumes without deploying additional servers—a limitation of the earlier two-server approach (KBC), which required extra infrastructure in high-density scenarios.

Third, our evaluation shows that this architectural redesign yields measurable performance gains without any new cryptographic algorithms. As reported in the revised manuscript, the integrated CBS consistently maintains authentication delays below the critical 100 ms VANET requirement, even in heavy traffic scenarios. By shortening the signal path and reducing duplicate work (e.g., cryptographic operations and database lookups performed separately by AS and TGS), the CBS can serve substantially more vehicle authentications per second.

Fourth, the CBS design continues to leverage blockchain for storing authenticators and does not alter the Kerberos trust model, thereby maintaining the same security guarantees as the baseline scheme (e.g., no secret keys exposed, tamper-proof audit trail).

In summary, the CBS integration streamlines the signaling path and reduces system complexity while maintaining the rigorous security of the Kerberos–Blockchain protocol. This innovative redesign provides a clear improvement over the state-of-the-art [6,8].

The Combined Server (CBS) proposed in this work introduces clear protocol-level innovations beyond the physical consolidation of the Authentication Server (AS) and Ticket Granting Server (TGS). In traditional Kerberos, authentication involves two distinct rounds: one with the AS to obtain a Ticket-Granting Ticket (TGT), followed by another with the TGS to request a Service Ticket (ST). In contrast, the CBS unifies these steps into a single exchange, wherein the combined entity performs both identity verification and service ticket issuance. This redesign reduces protocol rounds, minimizes communication overhead, and streamlines the client-side authentication flow.

In addition, the CBS enables inline ticket derivation, eliminating the need for intermediate credentials and redundant cryptographic operations. It also integrates a blockchain-based session validation mechanism, allowing for seamless handovers between Roadside Units (RSUs) without requiring full re-authentication. This protocol-level optimization enhances performance under VANET mobility constraints while preserving Kerberos security semantics.

In summary, the CBS constitutes a purposeful architectural and protocol-level redesign, not merely a colocation of servers, yielding measurable improvements in latency, signaling complexity, and mobility support.

The proposed CBS framework addresses the key limitations of the traditional KBS architecture by offering reduced delay, centralized key management, elimination of inter-module communication, and improved code maintainability, thereby providing a more robust and scalable solution for secure VANET authentication. The key difference in both scenario are shown in Table 3.

#### 5.2.1. Comparison of Vehicle Registration of AS in KBC vs. CBS

In the original Kerberos-based VANET design, vehicle registration and initial authentication are handled by the Authentication Server (AS) via a dedicated function, commonly referred to as handleVehicleRA. This routine processes a vehicle’s registration request by verifying the vehicle’s identity (e.g., via a pre-shared secret or digital certificate) and then issuing the necessary Kerberos credentials, namely a Ticket-Granting Ticket (TGT) and a session key [8]. Internally, the AS generates a random session key for vehicle–TGS communication. It then constructs the TGT containing this session key and vehicle identity, encrypting it with the TGS’s secret key. Simultaneously, the session key and accompanying metadata are encrypted with the vehicle’s secret key. The response sent to the vehicle therefore includes two encrypted segments: one encrypted for the vehicle, and the other (the TGT) encrypted for the TGS. This follows the standard Kerberos key exchange flow.

The encryption used in handleVehicleRA is based on AES-128, and both the TGT and the session key are handled through symmetric encryption. Each vehicle possesses a long-term secret key shared only with the AS, which is used to decrypt the session key and associated data. The TGT includes the session key and is readable only by the TGS, ensuring the integrity of the authentication process. This handleVehicleRA function is explained in Algorithm 1.
**Algorithm 1:** handleVehicleRA—AS Registration Process** Input:** Authentication request $\left[ID_{v},\;\mathrm{Nonce}\right]$ **Output:** Encrypted session key $K_{TGSse}$ and Ticket Granting Ticket TGT1 **if** not VerifyID($ID_{v}$) **then;**2  **return** error response;3 **end if**4 $K_{Vs}\leftarrow$
GetVehicleKey$\left(ID_{v}\right)$;5 $K_{TGSse}\leftarrow$
GenerateAES128Key();6 TGT← AES_Envrypt$\left(\left[\;ID_{v},\;K_{TGSse},\;TS_{TGT},\;LT_{TGT}\;\right],\;K_{TGSs}\right)$;7 $AT_{AStV}\leftarrow$ AES_Envrypt$\left(\left[\;K_{TGSse},\;TS_{TGT},\;LT_{TGT}\;\right],\;K_{Vs}\right)$;8 **return**
$\left[\;AT_{AStV},\;TGT\;\right]$;

In the proposed unified architecture, the CBS consolidates both AS and TGS functions into a single module. The core function responsible for this operation is handleRegistration OfVehiclePacket. Upon receiving a registration packet, the CBS verifies the vehicle’s identity and proceeds to generate a fresh session key. It then creates a ticket embedding the session key and relevant metadata (e.g., vehicle ID, timestamp, validity duration). This ticket is encrypted with the CBS’s own master key. The session key and related metadata are simultaneously encrypted using the vehicle’s long-term secret key. This handleRegistrationOfVehiclePacket function is explained in Algorithm 2.

Due to the merged roles, CBS can issue a final service ticket or authenticator directly to the vehicle, reducing the number of steps required in the authentication exchange. Additionally, the CBS logs a record of the authentication result onto a blockchain ledger, enabling subsequent verification by RSUs or other entities.
**Algorithm 2:** handleRegistrationOfVehiclePacket—CBS Registration Process **Input:** Registration packet $\left[ID_{v},\;\mathrm{credentials}\right]$ **Output:** Encrypted session key $K_{TGSse}$ and Ticket Granting Ticket TGT1 **if** not VerifyCreds($ID_{v},\;\mathrm{credentials}$) **then**;2  **return** error response;3 **end if**4 $K_{Vs}\leftarrow$
GetVehicleKey$\left(ID_{v}\right)$;5 $K_{TGSse}\leftarrow$
GenerateAES128Key();6 TGT← AES_Encrypt$\left(\left[\;ID_{v},\;K_{TGSse},\;TS_{TGT},\;LT_{TGT}\;\right],\;K_{TGSs}\right)$;7 $AT_{AStV}\leftarrow$ AES_Encrypt$\left(\left[\;K_{TGSse},\;TS_{TGT},\;LT_{TGT}\;\right],\;K_{Vs}\right)$;8 **return**
$\left[\;AT_{AStV},\;TGT\;\right]$;

For encryption and key handling, both functions apply AES-128 encryption to ensure secure session establishment, but they differ in mechanism and performance. In both implementations, a secure session key is generated by the server and distributed securely, encrypted for the vehicle using its own key, and stored in a ticket encrypted for the relevant server (TGS or CBS). The CBS implementation also benefits from blockchain integration, enabling verifiable and tamper-proof authentication logging. Overall, the unified CBS function achieves reduced latency, simpler protocol flow, and stronger encryption with AES, while preserving the secure key handling principles of Kerberos.

#### 5.2.2. Comparison of Authentication Ticket

In the Authentication Server (AS) implementation, the function aesEncrypt is utilized to secure the initial authentication ticket, referred to as the Ticket Granting Ticket (TGT), generated for the vehicle. After verifying the vehicle’s identity, the AS generates a fresh 128-bit Ticket Granting Server session key ($K_{TGSse}$), which is shared between the vehicle and the Ticket Granting Server (TGS). This session key, along with the vehicle’s identifier ($ID_{v}$), timestamp ($TS_{TGT}$), and requested ticket lifetime ($Req_{TGT}$), is encrypted using AES-128 with the TGS’s long-term secret key ($K_{TGSs}$) to produce the TGT. Additionally, the AS encrypts the same session key and its associated metadata using the Vehicle’s secret key ($K_{Vs}$) to securely deliver it to the On-Board Unit (OBU). This dual encryption ensures confidentiality and isolation of trust between the communicating entities. This aesEncrypt function is explained in Algorithm 3.

In contrast, the Combined Server (CBS) integrates the roles of both the AS and TGS. When a vehicle submits a request containing a TGT and the authenticator message ($AU_{VtTGS}$), the handleVehicleToTGSPacket function first decrypts the TGT using $K_{TGSs}$ to retrieve the embedded session key $K_{TGSse}$ and validate the ticket’s timestamp. The authenticator $AU_{VtTGS}$ is then decrypted using $K_{TGSse}$ to verify the freshness and authenticity of the request. Upon successful validation, the CBS generates a new 128-bit service session key ($K_{Sse}$) to enable secure communication with the target service. The generateTicket function then encrypts this session key along with the required metadata using the service’s secret key ($K_{Ss}$) to produce the Service Ticket (ST), which is returned to the vehicle along with the encrypted attribute ticket ($AT_{TGStV}$), both protected under AES-128 encryption with $K_{TGSse}$. This handleVehicleToTGSPacket function is explained in Algorithm 4.

Therefore, the CBS architecture enhances the authentication process by eliminating inter-server communication and combining validation and issuance operations within a single module. This design significantly reduces latency and signaling overhead, rendering it particularly well suited for dynamic and resource-constrained VANET environments.
**Algorithm 3:** Service Ticket Issuance-KBC **Input:** Received TGT, $AU_{VtTGS}$, requested $ID_{S}$ **Output:** Encrypted session key $K_{Sse}$ and Service Ticket ST 1 $TGT_{\mathrm{dec}}\leftarrow$ AES_Decrypt$\left(TGT,\;K_{TGSs}\right)$;2 $K_{TGSse}\leftarrow TGT_{\mathrm{dec}}.K_{TGSse}$;3 $AU_{\mathrm{dec}}\leftarrow$ AES_Decrypt$\left(AU_{VtTGS},\;K_{TGSse}\right)$;4 **if** (AU is valid **and** timestamp is fresh) **then**;5  $K_{Sse}\leftarrow$
GenerateAES128Key();6  ST← AES_Encrypt$\left(\left[\;ID_{v},\;ID_{S},\;K_{Sse},\;TS_{ST},\;LT_{ST}\;\right],\;K_{Ss}\right)$;7  $AT_{TGStV}\leftarrow$ AES_Encrypt$\left(\left[\;ID_{S},\;TS_{ST},\;LT_{ST},\;K_{Sse}\;\right],\;K_{TGSse}\right)$;8  **return**
$\left[\;ST,\;AT_{TGStV}\;\right]$;9 **end if**

**Algorithm 4:** Service Ticket Issuance—CBS **Input:** Received TGT, $AU_{VtTGS}$, requested service $ID_{S}$ **Output:** Encrypted session key $K_{Sse}$ and Service Ticket ST  1 $TGT_{\mathrm{dec}}\leftarrow$ AES_Decrypt$\left(TGT,\;K_{TGSs}\right)$;  2 $K_{TGSse}\leftarrow TGT_{\mathrm{dec}}.K_{TGSse}$;  3 $AU_{\mathrm{dec}}\leftarrow$ AES_Decrypt$\left(AU_{VtTGS},\;K_{TGSse}\right)$;  4 **if** ($AU_{\mathrm{dec}}$ is valid **and**
$TS_{VA}$ is fresh) **then**;  5  $K_{Sse}\leftarrow$
GenerateAES128Key();  6  ST← AES_Encrypt$\left(\left[\;ID_{v},\;ID_{S},\;K_{Sse},\;TS_{ST},\;LT_{ST}\;\right],\;K_{Ss}\right)$;  7  $AT_{TGStV}\leftarrow$ AES_Encrypt$\left(\left[\;ID_{S},\;K_{Sse},\;TS_{ST},\;LT_{ST}\;\right],\;K_{TGSse}\right)$;  8  **return**
$\left[\;ST,\;AT_{TGStV}\;\right]$;  9 **else**;10  **return** error response;11 **end if**

#### 5.2.3. Authentication Logic and Encryption Handling in KBC and TGS Functions

The handleVehicleToTGSPacket function in the CBS and the handlePacketFrom Vehicle function in the Ticket Granting Server (TGS) module perform distinct but complementary roles within the proposed authentication protocol. In the CBS stage, the handleVehicleTo TGSPacket function is responsible for verifying the vehicle’s identity using the Vehicle’s secret key ($K_{Vs}$). Upon successful validation, the CBS generates a fresh Ticket Granting Server session key ($K_{TGSse}$) for secure communication between the vehicle and the TGS. This session key is encapsulated in two outputs: (i) the Ticket Granting Ticket (TGT), encrypted with the TGS’s long-term secret key ($K_{TGSs}$), and (ii) the authentication ticket from KBC to vehicle ($AT_{AStV}$), encrypted with the vehicle’s secret key ($K_{Vs}$). The TGT is designed to be opaque to the vehicle and validated solely by the TGS, while the $AT_{AStV}$ enables the vehicle to retrieve the session key $K_{TGSse}$. The handleVehicleToTGSPacket function is explained in Algorithm 5.

In contrast, the handlePacketFromVehicle function explained in Algorithm 5 in the TGS processes authentication requests from vehicles by validating the previously issued TGT and the Authenticator from vehicle to TGS ($AU_{VtTGS}$). The TGT is decrypted using $K_{TGSs}$ to extract $K_{TGSse}$, which is then used to decrypt and validate $AU_{VtTGS}$. This dual-stage verification ensures both the integrity of the ticket and the freshness of the authentication request, thereby mitigating replay attacks. Upon successful validation, the TGS generates a Service session key ($K_{Sse}$) to be shared between the vehicle and the target service. This key is encapsulated in two forms: the Service Ticket (ST), encrypted with the service’s secret key ($K_{Ss}$), and the attribute ticket from TGS to vehicle ($AT_{TGStV}$), encrypted with $K_{TGSse}$ for decryption by the vehicle. Both the CBS and TGS modules thereby implement a layered encryption strategy to safeguard key confidentiality and mutual authentication using timestamped authenticators and rigorous key validation procedures tailored for secure operation in Vehicular Ad Hoc Networks (VANETs).
**Algorithm 5: **handlePacketFromVehicle—Service Ticket Issuance and TGT Verification (TGS) **Input:** Request $\left[TGT,\;AU_{VtTGS},\;AT_{VtTGS},\;ID_{S}\right]$ **Output:** Service Ticket ST and Access Ticket $AT_{TGStV}$  1 $TGT_{dec}\leftarrow$ AES_Decrypt$\left(TGT,\;K_{TGSs}\right)$;  2 Extract $\left[\;ID_{v},\;ID_{TGS},\;TS_{TGT},\;IP_{v},\;LT_{TGT},\;K_{TGSse}\;\right]$ from $TGT_{dec}$;  3 **if** (Expired$\left(TS_{TGT},\;LT_{TGT}\right)$
**or** malformed format) **then**;  4  **return** error (invalid or expired TGT);  5 **end if**  6 $AU_{dec}\leftarrow$ AES_Decrypt$\left(AU_{VtTGS},\;K_{TGSse}\right)$;  7 Extract $\left[\;ID_{v}^{\prime },\;TS_{VA}\;\right]$ from $AU_{dec}$;  8 **if** ($ID_{v}\neq ID_{v}^{\prime }$
**or** AU replayed **or** IP mismatch) **then**;  9  **return** error (invalid $AU_{VtTGS}$);10 **end if**11 Mark $AU_{VtTGS}$ as used;12 $K_{Sse}\leftarrow$
GenerateAES128Key();13 $AT_{plain}\leftarrow \left[\;ID_{S},\;TS_{TGStV},\;LT_{ST},\;K_{Sse}\;\right]$;14 $AT_{TGStV}\leftarrow$ AES_Encrypt$\left(AT_{plain},\;K_{TGSse}\right)$;15 $ST_{plain}\leftarrow \left[\;ID_{v},\;ID_{S},\;TS_{ST},\;IP_{v},\;LT_{ST},\;K_{Sse}\;\right]$;16 ST← AES_Encrypt$\left(ST_{plain},\;K_{Ss}\right)$;17 **return**
$\left[\;ST,\;AT_{TGStV}\;\right]$;

### 5.3. The System Phases

The phases of the proposed system are divided into five stages, including a system initialization and registration phase, vehicles and CBS communication stage, CBS and RSU interaction, an authentication message upon uploading to the blockchain, and a handover phase, which are shown in Figure 3.

#### 5.3.1. System Initialization and Registration Phase

During the offline registration phase, vehicles submit their credentials, including the vehicle ID, password, origin, destination, service type, and access rights (such as read, write, and modify permissions). Similarly, Roadside Units (RSUs) are registered with details like their physical location, MAC address, IP address, and RSU ID. Before the verification phase begins, the CBS generates and securely distributes secret keys to each entity involved.

#### 5.3.2. Vehicles and CBS Communication Stage

This phase includes communications that are relayed from the vehicle to CBS, and, conversely, reflecting the processes outlined in steps (a) and (b) illustrated in Figure 3. In the initial stage, the vehicle conveys a Request Authentication (RA) to the CBS, which comprises the $ID_{v}$, the particular service designation that the vehicle aims to access (in this scenario, the service designation is related to RSU service), $IP_{v}$, and the lifetime (LT). The LT limits the temporal span, thereby bolstering system security through a bounded time constraint. This information will be transmitted to the CBS.

The messages dispatched by the vehicles to the CBS are delineated in Equation (3). The corresponding response messages are represented in Equations (4), (5), and (7). The parenthesis symbol signifies a collection of unencrypted messages, whereas the bracket symbol represents the encrypted messages.

(3) $$\begin{array}{c} RA=(ID_{v}||ID_{s}||IP_{v}||Req_{LT}). \end{array}$$

(4) $$\begin{array}{c} AT_{VtTGS}=(ID_{s}||Req_{LT}). \end{array}$$

(5) $$\begin{array}{c} AU_{VtTGS}=K_{TGSse}[ID_{v}||TS_{VA}]. \end{array}$$

(6) $$\begin{array}{c} ST=K_{Ss}[(ST||ID_{v}||ID_{S}||TS_{TGStV}||IP_{v}||LT_{ST}\left|\right|K_{Sse})] \end{array}$$

The initial function of CBS involves maintaining a database of authenticated users along with their associated secret keys. When a verification request is received, the CBS checks whether the identifier $ID_{v}$ and corresponding message exist within this registry. If validation is successful, the secret key $K_{Vs}$ is retrieved. The CBS then generates an attribute token $AT_{VtTGS}$, which includes the service identifier $ID_{s}$, the requested lifetime (LT), and a specified validity period. This information is encrypted using a randomly generated symmetric session key $K_{TGSse}$, which is used by the user to decrypt subsequent communications from the CBS and RSU during the current session. Additionally, the attribute message $AT_{VtTGS}$ is encrypted with the vehicle’s secret key $K_{Vs}$. These encrypted messages are then transmitted from the CBS to the vehicle.

Following this, the CBS creates the authentication message $AU_{VtTGS}$, composed of the vehicle ID $ID_{v}$ and a timestamp $TS_{VA}$. This message is encrypted using the session key $K_{TGSse}$, employing AES encryption with a 128-bit key. Once the $AU_{VtTGS}$ is generated, it is uploaded to the blockchain network in step (c) for secure storage. Afterward, the same message is forwarded to the RSU in step (d). Further explanation of the blockchain storage mechanism for $AU_{VtTGS}$ is provided in Section 5.3.5.

#### 5.3.3. Vehicles and RSU Interaction

Once the vehicle receives the attribute message and the service ticket, the authentication process advances to steps (e) and (f), as illustrated in Figure 3. Using the session key $K_{TGSse}$, the vehicle decrypts the received $AT_{TGStV}$ to retrieve the service session key $K_{Sse}$. With this key, the vehicle constructs a new authentication message $AU_{VtS}$, which contains its identifier $ID_{v}$ and a timestamp $TS_{VtS}$. This message is then encrypted using $K_{Sse}$. Subsequently, the vehicle sends both the service ticket and the encrypted authentication message to the intended service provider, which, in this context, is the RSU. The structure and content of the messages transmitted by the vehicle to the RSU are outlined below:

(7) $$\begin{array}{ccc} ST= & K_{Ss}[(ST||ID_{v}||ID_{S}||TS_{TGStV}\left|\right| & IP_{v}||LT_{ST}\left|\right|K_{Sse})]. \end{array}$$

(8) $$\begin{array}{c} AU_{VtS}=K_{Sse}[ID_{v}||TS_{VtS}]. \end{array}$$

(9) $$\begin{array}{c} AU_{Sm}=K_{Sse}[ID_{v}||TS_{StV}]. \end{array}$$

The RSU follows a process analogous to that previously executed by the TGS. It begins by decrypting the service ticket (ST) using its private key $K_{Ss}$, through which it obtains the session key $K_{Sse}$ for further communication. This session key is then used to decrypt the vehicle’s authentication message $AU_{VtS}$. After successful decryption and validation, the RSU generates a new authentication response message, denoted as $AU_{Sm}$, which contains its own identifier $ID_{RSU}$ and a timestamp $TS_{StV}$, as formulated in Equation (9).

The generated $AU_{Sm}$ is transmitted back to the vehicle and decrypted using the session key $K_{Sse}$. Upon receipt, the vehicle performs two critical checks: it verifies that the service name included in the response matches the expected service, and it validates the freshness of the message by examining the timestamp $TS_{StV}$, thereby preventing replay attacks. Additionally, the vehicle updates its local cache mechanism. Upon the successful completion of mutual authentication between the vehicle and the RSU, the service ticket is securely stored in the vehicle’s cache for subsequent use in future sessions.

#### 5.3.4. CBS and RSU Interaction

The CBS and the RSU exhibit a non-direct engagement during the preliminary authentication stage as well as throughout the handover procedure. In the preliminary authentication stage, as depicted in Figure 3, it is evident that the CBS and the RSU do not engage in direct communication; nevertheless, their operational roles are interconnected. The CBS has first task to oversee the initial authentication of vehicles that seek services from the RSU within the confines of a secure network. The second responsibility of the CBS is to encompass the verification of vehicle identities, the issuance of Ticket Granting Tickets (TGTs), and the encryption of user credentials to ensure security. Subsequently, it is also responsible for issuing service tickets, which are utilized by vehicles to gain access to RSU services. The service server then validates these tickets to authenticate the client and facilitate authorized access to services.

During the handover phase, the CBS generates the $AU_{VtTGS}$, which serves as the essential credential for enabling seamless authentication continuity. This credential is securely uploaded to the blockchain, where access control and upload operations are governed by a predefined smart contract. The underlying smart contract, implemented in Solidity, defines parameters such as the entity’s name, unique identifier, and associated network designation. Only the CBS, designated as the trusted authority, is authorized to upload the $AU_{VtTGS}$ credential to the blockchain.

When a vehicle initiates a handover to a new RSU, the RSU retrieves the corresponding $AU_{VtTGS}$ by submitting its own entity name to the blockchain interface. Access is granted through a consensus-based validation mechanism that ensures the authenticity and integrity of the request. Upon successful verification, the transaction is committed to the blockchain as a new block, thereby allowing for the RSU to access the stored $AU_{VtTGS}$ and complete the handover process securely.

#### 5.3.5. Authentication Message Uploading in the Blockchain Phase

Once the $AU_{VtTGS}$ is generated by the CBS, it is subsequently stored on the blockchain. This credential serves as proof of the vehicle’s authenticity during future authentication attempts. The upload and retrieval of the $AU_{VtTGS}$ are facilitated through smart contract functions written in Solidity. Specifically, the off-chain simulation environment (OMNeT++) produces the $AU_{VtTGS}$, which must be transmitted through the smart contract interface before it can be visualized or interacted with on the Ganache platform. To store the authentication credential, the smart contract function AuthenticationMessageUploading() is invoked. This function accepts the $AU_{VtTGS}$ as an input argument, and a transaction request is initiated by the CBS to upload this data to the blockchain. Upon successful validation and mining of the transaction, the message is stored as part of a blockchain block. The function also emits events, which allow for observers to extract and verify the stored data via the Ganache user interface.

In this scenario, the CBS is modeled as the sender, while the RSU acts as the intended recipient. For identification purposes, the entity ID for the CBS is assigned as “1,” and the RSU is designated as “2.” Both entities operate within a shared blockchain network labeled “TsushimaVanet.” To retrieve the $AU_{VtTGS}$, the RSU sends a transaction request by calling the AuthenticationMessageAccessing() function within the smart contract. Once the transaction is confirmed, the RSU successfully obtains the $AU_{VtTGS}$ from the blockchain, enabling it to complete the authentication or handover operation securely.

#### 5.3.6. Handover Phase

When a vehicle exits an RSU’s coverage, a handover process begins with the source RSU verifying the target RSU. The vehicle sends a request, prompting the target RSU to upload an authentication message to the blockchain. After validation via smart contracts, the blockchain returns an authentication unit. The RSU confirms the vehicle’s data, completes the handover, and the vehicle updates and shares the new state.

### 5.4. Blockchain Solidity Algorithm

The blockchain component of the proposed authentication scheme is implemented using a smart contract deployed on the Ethereum network. This smart contract functions as a decentralized access control layer that governs which entities are permitted to upload and retrieve authentication credentials during the authentication and handover stages. Unlike conventional off-chain logging mechanisms, the Solidity-based contract ensures that all authentication messages are immutably stored and cryptographically verifiable, thereby preventing tampering or message forgery, even in high-mobility VANET scenarios.

We explained the pseudocode of Ethereum Vanet Smart Contract in the Algorithm 6. The smart contract encodes three core functionalities: entity registration, authentication message uploading, and authentication message retrieval. First, the Entity Registration() function enforces a strict admission policy by validating the identity and network origin of each participant before granting permission to interact with the blockchain. Only entities belonging to the “TsushimaVanet” domain are accepted, ensuring that unauthorized nodes cannot inject forged credentials into the ledger. Once registered, the Combined Server (CBS), acting as the Trusted Authentication Server (TAS), invokes the AuthenticationMessageUploading() function to store the authentication proof ($AU_{VtTGS}$) immutably on-chain. During a handover event, the RSU retrieves this credential by calling the AuthenticationMessageAccessing() function, which enables rapid re-authentication without re-contacting the CBS. This mechanism effectively offloads verification overhead from the CBS to the blockchain layer while leveraging Ethereum’s tamper-resistance and transparency to prevent replay or impersonation attacks. As a result, the smart contract not only acts as a persistence layer, but also as an access-control and trust anchor, ensuring that each authentication message remains verifiable, auditable, and cryptographically bound to a legitimate entity within the network.
**Algorithm 6:** EthereumVanet Contract Pseudocode 
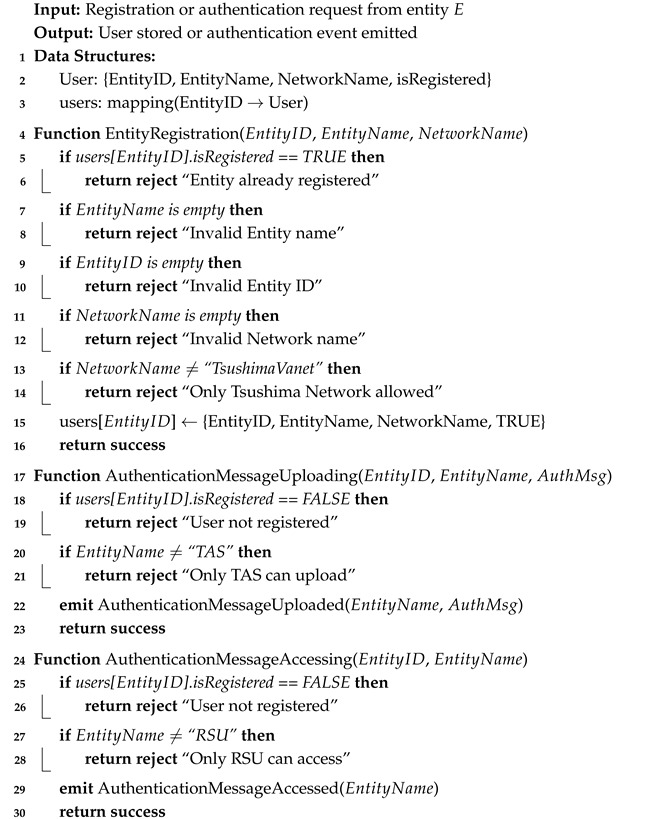


### 5.5. Real-World Deployment Options and Architectural Trade-Offs

In our architecture, the Combined Server (CBS) is deployed as an intermediate edge node positioned between multiple Roadside Units (RSUs), rather than being embedded within each RSU or centralized in a remote ITS cloud. This design choice reflects a trade-off strategy that balances latency, coverage, and fault tolerance.

By situating the CBS at a logical midpoint—topologically close to several RSUs, but not co-located—it can efficiently manage authentication requests from multiple RSUs within a cluster. This reduces the number of CBS instances required across a city-wide VANET while preserving low-latency communication paths to vehicles. Because CBS nodes are not centralized, the architecture avoids the high WAN latency and single-point-of-failure risks inherent in cloud-based deployments.

Furthermore, this semi-distributed design improves system resilience: if one CBS node fails, only the RSU cluster it serves is impacted, while the rest of the network remains operational. Compared to placing the CBS in each RSU (which increases deployment cost and redundancy), or centralizing it in a data center (which increases delay and vulnerability), this intermediate approach provides a practical compromise between scalability, performance, and fault isolation. This deployment strategy aligns with our OMNeT++ simulation, where the CBS was modeled as a virtual entity bridging the RSUs in the network.

## 6. Evaluation and Results

This section presents the performance analysis of the proposed CBS integrated with blockchain for VANET authentication. The evaluation is conducted using OMNeT++ and SUMO for network and mobility simulation, while blockchain functionality is implemented via Ganache and Truffle.

### 6.1. Evaluation

To assess the performance and practicality of the proposed authentication framework, a series of evaluations were designed across both on-chain and off-chain environments.

**Figure 5:**
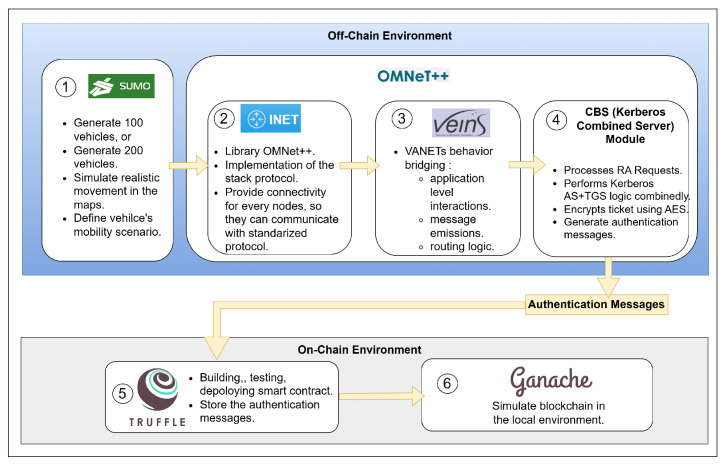
System environment diagram.

Figure 5 illustrates the implementation flow of the proposed VANET authentication system, which operates across two main environments: the Off-Chain Environment and the On-Chain Environment. The simulation begins with SUMO (Simulation of Urban Mobility), which generates 100 vehicles and simulates their realistic mobility patterns within an urban traffic map. These mobility traces are integrated into the INET framework within OMNeT++, which provides the standard network protocol stack (TCP, UDP, IPv4, and wireless communication) to enable data transmission between vehicles and Roadside Units (RSUs). VEINS acts as the bridge between SUMO and OMNeT++, supporting VANET behavior, message emission control, and routing logic. It also triggers each vehicle to send authentication requests (RA) upon entering the network.

The authentication request is processed by CBS, a custom module that integrates Kerberos AS and TGS functionalities along with AES-128 encryption. CBS generates the Authentication Message and transmits it to the On-Chain Environment, where Truffle manages the deployment of Ethereum smart contracts and Ganache simulates the local blockchain. The detail specification of the system environment is in Table 4.

**Figure 6:**
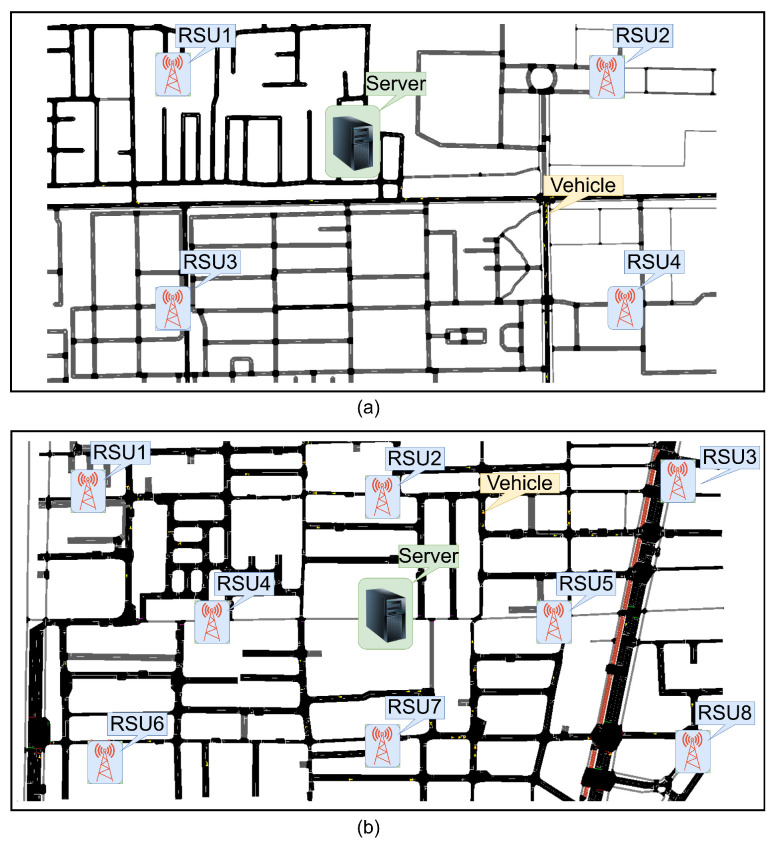
Maps for the scenario of (**a**) suburban and (**b**) urban area.

We conducted two scenarios to evaluate the system and compare the KBC approach with the CBS approach. The initial scenario concentrates on a suburban area. In this context, we use 100 vehicles, complemented by four RSUs, alongside a server. The experimental procedures were carried out utilizing the map representations of the Tsushima Campus area in Okayama, Japan, as depicted in Figure 6a. The maps were generated by OpenStreetMap, which operates under the open database license (ODbL) integrated with SUMO, which is characterized as a permissive open-source licensing framework.

In the second scenario, we increase the number of vehicles from 100 to 200 as a representation of the infrastructure in an urban area. This particular scenario was executed in Okayama Station area in Okayama City, Japan, as depicted in Figure 6b. The aim of this was to ascertain whether the efficacy of the authentication system remains within acceptable parameters as the vehicle count escalates. We positioned the server at the central locus of the maps. The diminutive yellow dots on the maps signify the vehicles. In light of the enhanced infrastructural availability within the urban context, we also doubled the quantity of Roadside Units (RSUs) from four to eight.

To prove our proposed system’s effectiveness, we assessed the parameters that we used in the testing scenarios, including authentication delay, throughput, and signaling overhead. Initially, the evaluation replicates the methodology of previous studies to enable a direct and accurate comparison of system performance. Subsequently, the number of vehicles was progressively increased, up to a maximum of 300 vehicles, to determine the system’s capacity while maintaining authentication delays within the acceptable thresholds defined for VANET environments.

### 6.2. The Performance Results

This section presents a detailed analysis of the simulation outcomes, with a focus on key network performance metrics. Specifically, it examines the transmission and processing delays associated with authentication messages, as well as the overall signaling overhead incurred by the system. To ensure a comprehensive evaluation, both suburban and urban traffic environments are modeled, enabling performance assessment under varying vehicular densities.

#### 6.2.1. Delay Analysis and Impact of the Protocol Architecture

**Figure 7:**
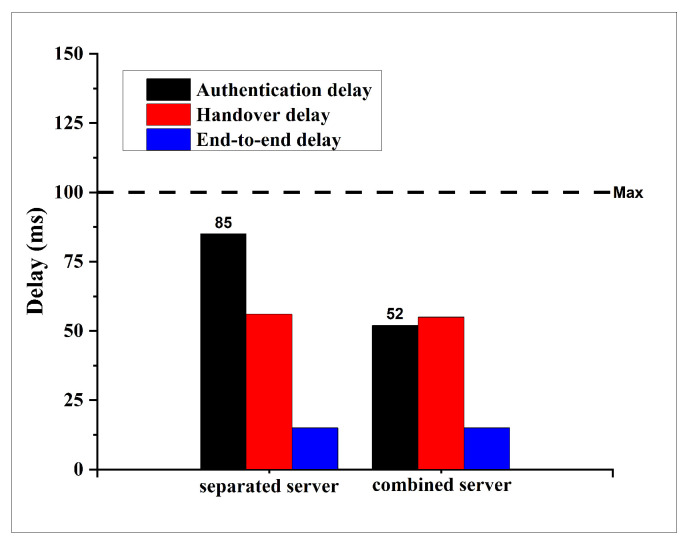
Effects of combined server on delays in suburban scenario (number of vehicles = 100).

**Figure 8:**
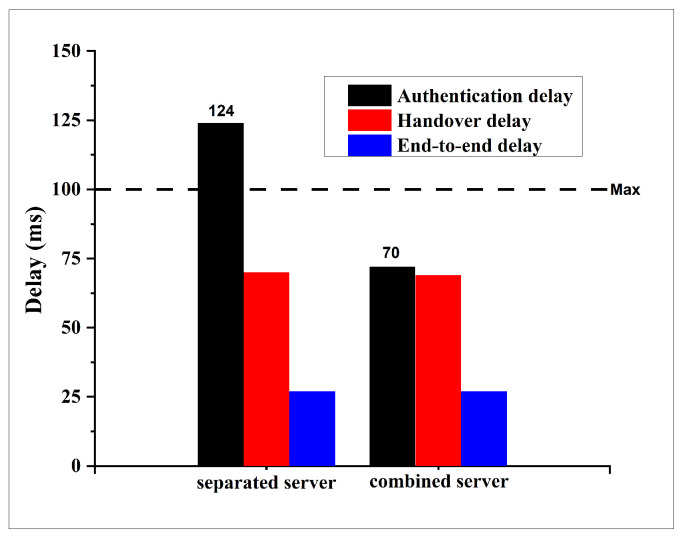
Effects of combined server on delays in urban scenario (number of vehicles = 200).

To evaluate the delay performance of the proposed Combined Server (CBS) protocol, we conducted a comparative analysis of three key delay metrics: authentication delay, handover delay, and end-to-end delay. These metrics were compared against those from the previously established Kerberos–Blockchain (KBC) system [8] under two different vehicular network scenarios: a suburban environment comprising 100 vehicles and an urban environment comprising 200 vehicles. The results are illustrated in Figure 7 for the suburban scenario and Figure 8 for the urban scenario.

In the suburban scenario, a notable reduction in authentication delay was observed, decreasing from 85 ms in the KBC approach to 52 ms in the CBS approach. In contrast, both the handover delay and the end-to-end delay remained unchanged at approximately 55 ms and 15 ms, respectively.

A similar pattern emerged in the urban scenario with 200 vehicles. The authentication delay in the CBS system was reduced from 124 ms to 70 ms, effectively bringing it below the commonly accepted VANET threshold of 100 ms. However, no significant differences were observed in the handover and end-to-end delays, which remained consistent with those recorded in the suburban setting.

Across both scenarios, the reduction in authentication delay highlights the effectiveness of the CBS protocol in improving initial authentication efficiency. Notably, the previous KBC protocol exhibited authentication delays that exceeded the acceptable upper bound for VANET applications, whereas the proposed CBS protocol successfully reduced the delay to within the required limits. This improvement is primarily attributed to the architectural simplification introduced in CBS, which consolidates the AS and TGS functionalities into a single server.

By collapsing the AS and TGS roles, simplified architectures reduce the number of message exchanges required during authentication. This architectural streamlining results in lower authentication delay. Gao et al. [30] demonstrated that using Proxy Mobile IPv6 (PMIPv6) in VANETs reduces authentication delay during handovers due to localized mobility anchors. Similarly, Wang et al. [31] showed that the SREHA handover scheme reduces delay by removing redundant validation steps. These results indicate that fewer authentication stages translate into faster and more efficient processes.

However, the observed improvements are limited to the authentication delay metric. The handover and end-to-end delays remain unaffected, as the modifications introduced in the CBS target only the initial authentication process and do not alter the mechanisms responsible for handover or data forwarding operations. It is noteworthy that the measured handover delay remains below the critical authentication delay threshold. Furthermore, the observed end-to-end delay consistently falls within the acceptable range defined by the ETSI TS 122 186 standard, which mandates that end-to-end delays for safety-related applications should not exceed 20 ms [32].

#### 6.2.2. The Scalability Challenge

To evaluate the scalability limitations of the proposed system, we conducted additional experiments by incrementally increasing the number of vehicles from 100 to 400. In this evaluation, we focused exclusively on authentication delay, as previous results indicated that handover delay and end-to-end delay remained unaffected by changes in network density.

**Figure 9:**
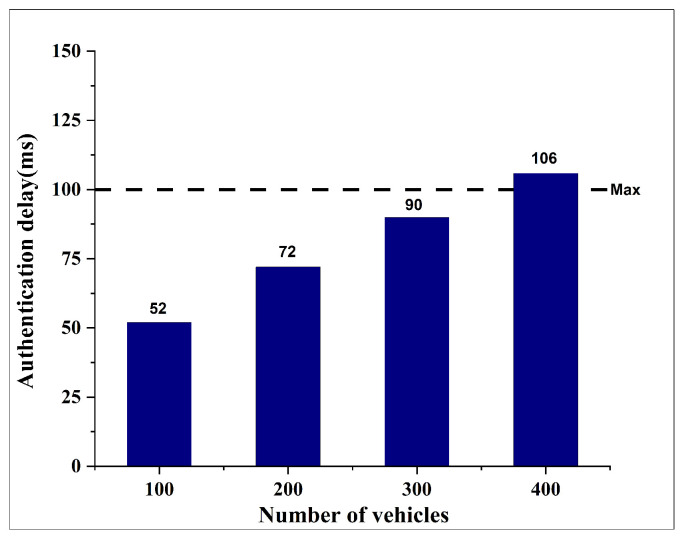
Effects of vehicle number on authentication delays in combined server urban scenario (up to 400 vehicles).

As illustrated in Figure 9, the authentication delay remains below the acceptable threshold of 100 ms for up to 300 vehicles. However, when the number of vehicles reaches 400, the authentication delay exceeds the standard VANET threshold, increasing to 105.87 ms. This result indicates that while the proposed system demonstrates strong performance under moderate traffic conditions, its scalability may be constrained under high-load scenarios.

The increase in authentication delay beyond 400 vehicles is not caused by the blockchain component, but rather by congestion in the off-chain communication path of the VANET. When the number of vehicles increases beyond this threshold, the wireless channel becomes saturated and packet collisions begin to occur more frequently, causing repeated retransmissions and queueing in the MAC layer. As a result, the Combined Server (CBS) experiences a longer waiting time to receive authenticators from RSUs, which increases end-to-end delay even though the cryptographic computation time remains constant.

The reason performance is still stable below 300 vehicles is because the channel load remains within the contention window that the MAC scheduler can still handle without excessive backoff. In this regime, the RSUs can still forward authenticators to the CBS with relatively low interference, and the queuing delay remains negligible. However, once the number of active authentication requests approaches 350–400 vehicles, the medium access load surpasses the threshold where collisions begin to dominate. This shifts the bottleneck from computation to channel availability: even though the CBS remains computationally capable, it is starved of timely packets due to increasing retransmission overhead at the RSU layer. Therefore, 400 vehicles is where the VANET transitions from under-saturated to saturated channel conditions, which explains why the delay sharply rises above 100 ms only after this point.

Importantly, this degradation is unrelated to the blockchain layer. As reported in our previous work [8], the smart contract functions require only 29,733 GWEI and 27,208 GWEI, respectively, and the memory footprint remains below 30 kB, which is only a small fraction of typical block capacity. Therefore, the congestion observed at 400 vehicles is a networking-layer scalability issue rather than a ledger-side throughput limitation.

To mitigate this, the CBS architecture can be extended through horizontal scaling or regional load-balancing. Instead of a single CBS handling all vehicles in a large coverage area, multiple CBS instances (or shard-like clusters) can be deployed and assigned per-region or per-RSU group. This reduces contention by distributing authentication load across multiple servers while still preserving consistency through shared blockchain logging. Another mitigation strategy is to offload filtering and pre-verification to edge RSUs before forwarding requests to the CBS, so only valid and rate-limited authentication attempts reach the server. These strategies allow for the architecture to maintain sub-100 ms authentication delay, even at higher densities beyond 400 vehicles.

#### 6.2.3. Signalling Overhead

Signaling overhead refers to the control-related messages required to manage sessions, such as authentication, synchronization, and mobility updates. While essential, excessive signaling can consume bandwidth and processing time, reducing overall efficiency. In latency-sensitive environments like VANETs, minimizing signaling overhead is crucial to ensure faster response times and optimal resource usage. These signaling messages are not the main data (payload), but supporting messages including authentication protocol.

**Figure 10:**
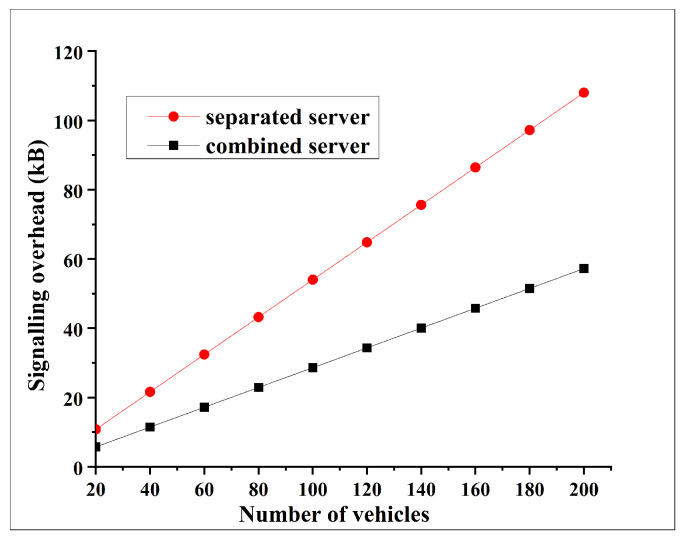
Comparison of signaling overhead between separated server and combined server.

We compare the signaling overhead from our proposed method (CBS) with the former method, KBC. The signaling overhead that we take is counted in every increasing 20 number of vehicles of both approach. The signaling overhead for this proposed system is calculated in Equation (10):

(10) $$\begin{array}{c} C_{KBC}=hop_{RSU-RSU}\cdot \left[a\cdot Trans_{u}\cdot L_{msg}\right] \end{array}$$

where $hop_{RSU-RSU}$ denotes the average distance between adjacent Roadside Units (RSUs), *a* represents the weight coefficient assigned to a specific communication link, $Trans_{u}$ refers to the transmission unit, and $L_{msg}$ indicates the total size of the messages exchanged during the signaling process. The message sizes used in the signaling evaluation for this study are summarized in Table 5, while the comparative analysis of signaling overhead with existing approaches is presented in Figure 10.

Figure 10 illustrates the comparative results of signaling overhead for both methods across increasing vehicle densities, with increments of 20 vehicles per simulation run. The Combined Server (CBS) consistently demonstrates a reduced signaling overhead compared to the Separated Server configuration used in the traditional KBC system. For instance, at 100 vehicles, the signaling overhead for KBC (AS + TGS) is approximately 54,000 bytes, while for CBS, it is only about 28,600 bytes. This trend continues across all tested scenarios, indicating that the CBS introduces a more efficient signaling mechanism.

The reduction in overhead is attributed to the architectural integration AS and TGS functionalities into a single server. This consolidation eliminates the need for inter-server communication, thus removing several control exchanges that were previously necessary in the KBC architecture. By simplifying the message flow and reducing redundancy, CBS minimizes the bandwidth consumption and processing delay associated with signaling, making it more suitable for high-density VANET environments.

It is important to note that the signaling overhead comparison in this study is conducted for up to 200 vehicles. This limitation is intentional, as the baseline KBC framework (separated server design) only supports simulations up to 200 vehicles. To ensure a fair and direct comparison, the signaling overhead results for CBS were also calculated within the same range. The reported 45% reduction in signaling overhead is derived by comparing the total overhead of the CBS against the combined overhead of the AS and TGS in the separated configuration, i.e.,

$$\mathrm{Improvement}(\%)\;=\frac{(\mathrm{AS}\;+\;\mathrm{TGS})-\mathrm{CBS}}{\left(\mathrm{AS}\;+\;\mathrm{TGS}\right)}\times 100.$$

This explicit comparison ensures that the percentage improvement is computed under equivalent conditions, providing a fair and consistent evaluation baseline.

Overall, the proposed CBS method achieves a substantial reduction in signaling overhead, enhancing the protocol’s scalability and responsiveness. This improvement is particularly critical in scenarios involving frequent mobility handovers and real-time authentication requirements [33].

#### 6.2.4. Throughput

We evaluate the throughput performance of a KBC protocol [8] under two different server architectures: a *separated server configuration* with distinct AS and TGS, and a *combined server implementation*, wherein a single CBS fulfills both AS and TGS roles. Experiments were conducted for two network sizes (100 vehicles and 200 vehicles) to observe how each architecture scales with the number of vehicles. The throughput (in bits per second, bps) was measured at the authentication servers, and we recorded the average sustained throughput as well as the minimum and maximum observed values over the measurement period for each scenario. Table 6 summarizes the results, comparing the separated and combined configurations for 100 and 200 vehicles.

As shown in Table 6, the Combined Server (CBS) consistently outperforms the Separated Server configuration (AS + TGS) across all tested scenarios. For the 100-vehicle case, the CBS achieves an average throughput of 627 bps, approximately double the combined throughput of AS (52 bps) and TGS (261 bps). The maximum CBS throughput reaches 4316 bps, far exceeding the peak values of the separated servers. Similarly, for the 200-vehicle case, the CBS maintains an average of 588 bps compared to a combined 343 bps in the separated setup. The improvement ratio ranges from about 70% to over 1100%, demonstrating the scalability and efficiency of the integrated CBS architecture. This performance gain results from the elimination of inter-server communication delays and optimized message handling within a unified authentication framework.

This stark difference underscores the efficiency advantage of the integrated approach. Several factors contribute to the higher throughput observed with the combined CBS architecture. First, merging the AS and TGS roles eliminates the inter-server communication overhead inherent in the traditional Kerberos workflow [8]. In the separated design, each vehicle’s authentication involves at least two sequential steps (AS followed by TGS), with network messages and processing delays between them. The CBS design removes this network round-trip between servers, reducing latency and CPU overhead per authentication request. With fewer context switches and message-passing delays, the server can complete each authentication transaction faster, effectively handling more requests per second.

Second, the processing is more *streamlined* in the combined implementation. Since a single server handles the entire authentication ticket issuance process, it can optimize the workflow internally (for example, by reusing cryptographic contexts or combining database lookups), rather than each server performing its own set of operations from scratch. This streamlining minimizes duplicated work and benefits overall throughput.

Third, resource utilization is improved through *resource consolidation*. Instead of two separate servers each running at partial load, the CBS concentrates computational resources into servicing a unified queue of requests. This consolidation ensures that the server’s full capacity is leveraged for the combined task, avoiding the scenario where one server might be idle while the other is a bottleneck.

We also calculate the statistic-related parameters from the throughput, including variance, standard deviation, and mean, in order to quantify not only the average performance, but also the degree of fluctuation under different traffic conditions. The variance indicates how far the throughput values deviate from the average, while the standard deviation provides a more interpretable measure of dispersion using the same unit as the original data. These metrics allow us to evaluate the stability of each architectural component (AS, TGS, and CBS) rather than relying solely on mean throughput.

The mean throughput μ for a dataset of *N* throughput samples $\left\{x_{1},x_{2},\ldots ,x_{N}\right\}$ is calculated as

(11) $$\mu =\frac{1}{N}\sum_{i=1}^{N}x_{i}$$

The variance $\sigma^{2}$, which measures the average squared deviation from the mean, is computed as

(12) $$\sigma^{2}=\frac{1}{N}\sum_{i=1}^{N}{\left(x_{i}-\mu \right)}^{2}$$

The standard deviation σ is then obtained as the square root of the variance:

(13) $$\sigma =\sqrt{\sigma^{2}}$$

By applying these equations to the measured throughput, we observe that the AS consistently exhibits low variance and standard deviation across all vehicle densities, indicating stable performance. In contrast, the TGS shows significantly higher variance that grows with increasing load, confirming that the instability originates primarily from the ticket issuance stage. This statistical evidence further supports the architectural motivation for the CBS, which reduces the fluctuation observed in the separated AS–TGS model.

The throughput variance and standard deviation results clearly reveal the different stability characteristics of the AS and TGS components under the separated-server architecture. For the 100-vehicle scenario, the AS exhibits very low fluctuation (variance = 10.64; standard deviation = 3.26), confirming that this stage of the authentication pipeline remains stable, even under concurrent request conditions. In contrast, the TGS already shows substantially higher dispersion (variance = 696.32; standard deviation = 26.39), indicating that most of the instability in the system originates from the ticket issuance process rather than the initial authentication step.

When the number of vehicles increases to 200, this behaviour becomes even more pronounced. While the AS variance remains small (rising only from 10.64 to 45.09), the TGS variance nearly doubles (from 696.32 to 1310.70), and its standard deviation increases from 26.39 to 36.20. This demonstrates that the TGS is far more sensitive to higher vehicle density and request concurrency than the AS. The degradation is therefore not uniformly distributed across the authentication pipeline, but rather is disproportionately concentrated in the TGS component.

These observations imply that the separated AS–TGS architecture is structurally imbalanced: the AS maintains stability as the system scales, while the TGS accumulates instability because it must perform ticket generation, validation, and partial storage operations during every session establishment. As a result, the TGS becomes the dominant source of throughput unpredictability, especially in high-mobility settings where requests arrive in short bursts rather than at uniform intervals.

This statistical behaviour explains the rationale for the proposed Combined Server (CBS) architecture. By consolidating the AS and TGS roles into a single execution unit and eliminating cross-server message exchange, the CBS mitigates the fluctuation patterns that previously emerged at the TGS boundary. In this design, variance and standard deviation do not merely serve as auxiliary metrics, but rather provide direct architectural evidence that instability is tied to the legacy separation of authentication stages.

For clarity and fairness of comparison, all reported improvement values (including the 104% throughput gain and the 45% signaling overhead reduction) are calculated against the baseline of the traditional separated-server model (AS + TGS), which reflects current Kerberos-based VANET deployments. AS-only or TGS-only configurations are not used as baselines because neither of them alone represents a complete authentication workflow. The chosen baseline therefore reflects the state-of-practice rather than a partial or idealized configuration. This clarification has now been explicitly added to Section 5.3 of the revised manuscript to ensure transparency in the interpretation of the performance gains achieved by the CBS architecture.

#### 6.2.5. Blockchain Latency and Computational Overhead Justification

Public blockchains such as Ethereum introduce non-trivial latency due to consensus protocols and block confirmation times. However, in the CBS architecture, blockchain does not participate in the time-critical portion of the authentication workflow. All identity verification, session key establishment, and ticket generation are executed off-chain by the Combined Server (CBS), while the blockchain is used only to store a hashed reference of the authentication result for auditability. Therefore, the latency measurements reported in this paper focus on the off-chain communication path (simulated in OMNeT++), which is the dominant factor in vehicular authentication responsiveness. The on-chain component is deliberately decoupled from real-time authentication, preventing consensus delay from affecting session establishment.

Since the blockchain layer in CBS is functionally identical to our previous Kerberos–Blockchain integration [8], its gas cost and storage footprint remain unchanged and do not require re-evaluation. In our earlier measurements [8], the *AuthenticationMessageUploading()* function consumed 29,733 GWEI, while *AuthenticationMessageAccessing()* consumed 27,208 GWEI, both significantly below the block gas limit of common Ethereum-based permissioned deployments. Furthermore, the maximum memory required to store 200 authentication records was only 29,894 bytes, far lower than the typical 1–2 MB blockchain block size. These results confirm that the storage and execution overhead of the blockchain layer is lightweight and does not hinder scalability.

Because this work introduces improvements exclusively in the off-chain authentication path—without modifying the smart contracts or ledger-side logic—re-running the blockchain benchmark would reproduce the same results and would not yield new insights. For this reason, we explicitly reference our previously validated blockchain performance results rather than duplicating experimental evaluation. This ensures continuity with prior work, while clarifying that the main contribution of the CBS lies in reducing authentication delay and improving privacy-preserving verification without increasing on-chain cost.

#### 6.2.6. The Impact of Mobility Patterns

In our performance analysis, we also examined how mobility patterns, specifically node density and vehicle speed, impact the CBS scheme’s effectiveness. Table 7 summarizes the findings across suburban and urban scenarios. Doubling the network size from 100 to 200 vehicles (medium to high density) modestly increased the CBS’ authentication delay (from ∼52 ms to ∼70 ms), keeping it below the 100 ms safety limit, whereas the legacy KBC architecture’s delay grew from 85 ms to 124 ms (violating the 100 ms threshold). This demonstrates that higher vehicle density (urban scenario) imposes a heavier load, yet the integrated server architecture maintains acceptable latency, unlike the separated server (KBC) system.

The estimated vehicle speeds presented in Table 7 (i.e., 30 km/h for urban and 60 km/h for suburban environments) are based on typical real-world traffic patterns and are supported by existing simulation studies. For instance, in [34], both urban and highway scenarios were modeled using SUMO and OMNeT++ with a maximum vehicle speed set to 20 m/s (approximately 72 km/h), serving as a general upper bound for VANET simulations. In our case, the speed estimates reflect the average effective speed observed under different node densities and mobility constraints in the SUMO-based Duaroute configuration, where urban layouts naturally induce slower movement due to higher densities and shorter road segments, while suburban scenarios allow for moderately higher speeds. These realistic estimations help us to contextualize the authentication delays achieved by our CBS architecture under varying mobility conditions.

### 6.3. Security Analysis and Threat Model of CBS Architecture

This subsection presents the security and threat analysis of the proposed Combined Blockchain Server (CBS) by formalizing the attacker capabilities and mapping them to classical VANET threats. We adopt a Dolev–Yao-style adversary, where the attacker can intercept, replay, or modify packets, impersonate entities, or attempt to tamper with authentication logs. However, the attacker is still bounded by cryptographic hardness assumptions and cannot break AES-128 or SHA-3 hashing in practical time.

The CBS architecture mitigates these threats through secure offline identity provisioning, which ensures that each vehicle and RSU is bound to a pre-distributed long-term secret key before it joins the network. During runtime, authentication uses ephemeral AES-128 session keys and timestamp-based freshness validation, preventing replay and impersonation. Since real identities are never transmitted in plaintext and are not stored on-chain, user privacy is preserved, even under a global passive adversary. Integrity of authentication records is guaranteed through blockchain immutability, while smart contracts enforce role-based access control so that only the CBS can upload authentication proofs and only authorized RSUs can retrieve them. Therefore, the blockchain layer strengthens accountability without exposing sensitive identity information.

The Table 8 summarizes the major VANET threats considered in this work and the corresponding mitigation techniques implemented by the CBS.

In summary, the CBS architecture provides confidentiality, integrity, and authentication guarantees while preventing replay, impersonation, and tampering attacks under classical VANET threat conditions. Since the design preserves privacy and avoids plaintext identity exposure, it mitigates both traffic analysis and tracking attacks without weakening the trust guarantees of Kerberos. This confirms that the CBS achieves stronger security properties while retaining low authentication latency suitable for large-scale VANET deployments.

Moreover, to ensure resilience under blockchain congestion, the architecture was evaluated and theoretically analyzed under conditions simulating variable blockchain load. Blockchain congestion can delay block confirmation and affect systems that rely on on-chain verification. In the proposed Combined Blockchain–Kerberos Server (CBS), this effect is minimized because the blockchain is used only for storing compact authentication logs, rather than performing the authentication itself. In the CBS, the blockchain layer only records compact verification hashes and timestamp logs instead of full authentication payloads. This design inherently minimizes on-chain data volume and reduces dependency on transaction confirmation speed. Thus, congestion does not interrupt or delay vehicle authentication in real time. The CBS incorporates a lightweight transaction-queue management mechanism in which the Authentication Log Contract (ALC) prioritizes low-gas, high-priority entries corresponding to recent authentication events. Vehicles receive authentication tickets immediately after Kerberos verification, while blockchain logging proceeds asynchronously. This mechanism ensures that authentication evidence remains tamper-proof and recoverable, even under heavy blockchain load.

### 6.4. Practical Deployment Feasibility: Cost, Energy, and Congestion Robustness

While the statistical evaluation demonstrates that the CBS significantly improves stability compared to the separated AS–TGS architecture, a complete assessment of real-world viability must also consider deployment cost, energy requirements, and behaviour under blockchain congestion. These factors determine whether the architecture can be feasibly deployed at scale in vehicular environments, particularly at RSU or edge nodes where hardware resources are limited and power consumption is an operational constraint.

From a hardware perspective, the CBS model is more economical than the traditional separated-server design because it eliminates the need to operate two independent authentication components. Instead of provisioning both AS and TGS as separate physical or virtual nodes, the CBS consolidates the functionality into a single execution unit. A low-cost embedded-class edge device, such as a Raspberry Pi 4 (USD 70–150) or an Intel NUC-class mini PC (USD 300–500), is already sufficient to host the merged Kerberos logic while maintaining the throughput observed in the evaluation. Therefore, the CBS not only reduces latency, but also lowers capital expenditure by reducing the number of deployed nodes.

Energy consumption also benefits from this consolidation. In the traditional AS + TGS architecture, both servers must remain continuously active, each incurring its own idle and peak power draw. The CBS reduces this baseline energy footprint by collapsing the authentication path into a single processing unit, thus preventing duplicate standby power and unnecessary inter-node signaling. Embedded servers suitable for RSU deployment typically operate within a 10–25 W envelope under load, while maintaining AS and TGS separately would require approximately 20–40 W cumulatively. This translates into a practical reduction in power usage by approximately 40–50% in realistic edge deployments.

In addition to hardware and energy benefits, the CBS also improves resilience under blockchain congestion. The separated AS–TGS model requires more frequent and sequential interactions with the blockchain, which amplifies performance degradation when the ledger experiences delayed confirmations or backlog. The CBS, on the other hand, maintains authentication state locally and reduces blockchain dependency to a single update per session, allowing for handovers to proceed, even during temporary congestion. This decoupling of responsiveness from on-chain transaction throughput ensures that temporary spikes in blockchain latency do not interrupt vehicular authentication, thereby improving operational robustness without sacrificing auditability.

Collectively, these properties demonstrate that the CBS architecture not only enhances computational stability, but also offers practical deployability under real-world constraints. Its lower hardware footprint, reduced power consumption, and tolerance to blockchain congestion make it well-suited for RSU-level or roadside edge deployment in large-scale VANET environments.

## 7. Conclusions

In this paper, we present an integrated authentication server architecture for VANET security that unifies the Authentication Server (AS) and the Ticket Granting Server (TGS) components of a Kerberos–Blockchain authentication system into a single Combined Blockchain Server (CBS). Our comprehensive evaluation implemented and tested the proposed system in simulated VANETs across both suburban and urban environments using Omnet++ integrated with SUMO traffic simulator, while the blockchain component was implemented on the Ganache platform. Most critically, this proposal successfully maintains authentication delays well below the 100 ms threshold required for VANET operations, even in high-density urban scenarios, while eliminating the need for additional infrastructure as vehicle volumes increase. The integrated architecture achieved approximately 104% higher throughput and 45% lower signaling overhead compared to the original separated server approach. Furthermore, by addressing critical authentication delay requirements while enhancing scalability, our approach contributes significantly to the practical implementation of secure and efficient VANETs for next-generation Intelligent Transportation Systems.

For future work, we plan to further enhance our current CBS authentication to address the unique challenges of ultra-dense urban VANET environments. This includes integrating lightweight cryptographic primitives specifically optimized for vehicular networks to further reduce authentication latency during high-traffic scenarios such as rush hours and major public events. Additionally, we aim to strengthen the security robustness of our system through VANET-specific security enhancements, including proof-of-location mechanisms, to prevent position spoofing attacks.

## Figures and Tables

**Table 2:** Annotations and abbreviations.

Entities		
Vehicular Ad Hoc Network		VANET
On-Board Unit		OBU
Vehicle		V
Trusted Authority Server		TAS
Authentication Server		AS
Ticket Granting Server		TGS
Roadside Unit		RSU
Keys		
Vehicle’s secret key		$K_{Vs}$
TGS secret key		$K_{TGSs}$
TGS session key		$K_{TGSse}$
Service session key		$K_{Sse}$
Service secret key		$K_{Ss}$
Signature of the Client		$P_{v}$
Signature KDC		$P_{kdc}$
Certificate KDC		$C_{kdc}$
Name of Message		
General	Request authentication	RA
	Reply	RP
	Ticket Granting Ticket	TGT
	Service Ticket	ST
Authentication msg	Vehicle auth V to TGS	$AU_{VtTGS}$
	Vehicle auth V to Service	$AU_{VtS}$
	Service auth message	$AU_{Sm}$
	Service authentication Message	FA
Attributes	Attributes AS to V	$AT_{AStV}$
	Attributes V to TGS	$AT_{VtTGS}$
	Attributes TGS to V	$AT_{TGStV}$
Message Contents		
ID	Vehicle’s ID	$ID_{v}$
	Service name/ID	$ID_{S}$
	TGS name/ID	$ID_{TGS}$
IP	User IP Address	$IP_{v}$
Req	Request lifetime for TGT	$Req_{TGT}$
	Request lifetime for ticket	$Req_{LT}$
Timestamp	Timestamp attributes AS to V	$TS_{AStV}$
	Timestamp TGT	$TS_{TGT}$
	Timestamp Vehicle authentication	$TS_{VA}$
	Timestamp attribute TGS to V	$TS_{TGStV}$
	Timestamp ST	$TS_{ST}$
	Timestamp vehicle to service	$TS_{VtS}$
	Timestamp service auth message	$TS_{StV}$
Parameter	DH parameter	$DH_{p}$
Lifetime	Lifetime for TGT	$LT_{TGT}$
	Lifetime for Service Ticket	$LT_{ST}$

**Table 3:** Comparison of key functional components between AS–TGS in the KBC [8] and CBS method.

Functionality	AS in KBC	TGS in KBC	CBS Method
Vehicle Registration	handleVehicleRA	Not Applicable	handleRegistrationOfVehiclePacket
Authentication Ticket	aesEncrypt	Not Applicable	generateTicket, handleVehicleToTGSPacket
Ticket Validation	Not Applicable	handlePacket FromVehicle	handleVehicleToTGSPacket
Key Management	Separate for AS	Separate for TGS	Unified in generateSecretKey, handleRegistrationOfVehiclePacket
Communication Overhead	Requires AS–TGS communication	Requires AS–TGS communication	Eliminated due to integration

**Table 4:** Implementation environment.

Software/Hardware	Configuration/Version
Operating System	Windows 11 22H2 64-bit
Processor	AMD Ryzen 7 5800U @ 1.90 GHz
RAM	16 GB
Truffle Framework	Version 5.11.0
Ganache (Blockchain Simulator)	Version 2.7.1
OMNeT++	Version 6.0.2
SUMO (Simulation of Urban MObility)	Version 1.4.0

**Table 5:** Message size in the signaling process.

Message Parameter	Size (Bytes)
Session Key	16
Vehicle ID	8
Timestamp (TS)	4
Ticket for Initial Authentication	8
HMAC	8
AES Input Bit Length	16
Lifetime	3
IP Address	16
Service Name	3

**Table 6:** Throughput comparison between Separated Server (KBC) [8] with Combined Server (CBS).

Vehicles	Separated Server (bps)			Combined Server (bps)	Improvement (%)
	AS	TGS	Total		
*100 Vehicles*					
Avg	51.95	261.0	312.95	627	100.3
Max	58.48	294.0	352.48	4316	1124.5
Min	38.52	58.0	96.52	501	419.1
Variance	10.64	696.32		167,690.25	
Standard Deviation	3.26	26.39		409.5	
*200 Vehicles*					
Avg	56.00	287.0	343.0	588	71.4
Max	108.21	545.6	653.81	4316	560.1
Min	52.35	59.52	111.87	458	309.4
Variance	45.09	1310.7		174,991.48	
Standard Deviation	36.2	26.39		418.3	

**Table 7:** CBS performance across mobility scenarios.

Scenario	Density	Authentication Delay	Speed
Suburban (Medium Density)	100 nodes	52 ms	Moderate (∼60 km/h)
Urban (High Density)	200 nodes	69.99 ms	Low (∼30 km/h)

**Table 8:** Threat-mitigation mapping for CBS architecture.

Threat	Impact	Mitigation in CBS
Impersonation	Unauthorized access	Offline provisioning + long-term secret keys
Replay Attack	Unauthorized reuse of old tokens	Nonces and timestamps in authenticators
MITM Attack	Message alteration in transit	End-to-end symmetric encryption
Tampering/Ledger Rewrite	Manipulation of logs	Blockchain immutability (SHA-3 + chained blocks)
Unauthorized Access	Exposure of auth records	Smart-contract role-based access
DoS	Resource exhaustion	On-chain caching + throttling
Key Compromise	Session leakage	Forward secrecy via ephemeral keys

## Data Availability

Data are contained within the article.
